# Genetic and environmental dissection of biomass accumulation in multi-genotype maize canopies

**DOI:** 10.1093/jxb/ery309

**Published:** 2019-04-18

**Authors:** Tsu-Wei Chen, Llorenç Cabrera-Bosquet, Santiago Alvarez Prado, Raphaël Perez, Simon Artzet, Christophe Pradal, Aude Coupel-Ledru, Christian Fournier, François Tardieu

**Affiliations:** 1 Université de Montpellier, INRA, LEPSE, Montpellier, France; 2 CIRAD, UMR AGAP, Montpellier, France

**Keywords:** canopy heterogeneity, GWAS, high-throughput phenotyping, inter-genotypic competition, light interception, light use efficiency, maize, plant architecture, varietal mixture

## Abstract

Multi-genotype canopies are frequent in phenotyping experiments and are of increasing interest in agriculture. Radiation interception efficiency (RIE) and radiation use efficiency (RUE) have low heritabilities in such canopies. We propose a revised Monteith equation that identifies environmental and genetic components of RIE and RUE. An environmental term, a component of RIE, characterizes the effect of the presence or absence of neighbours on light interception. The ability of a given plant to compete with its neighbours is then identified, which accounts for the genetic variability of RIE of plants having similar leaf areas. This method was used in three experiments in a phenotyping platform with 765 plants of 255 maize hybrids. As expected, the heritability of the environmental term was near zero, whereas that of the competitiveness term increased with phenological stage, resulting in the identification of quantitative trait loci. In the same way, RUE was dissected as an effect of intercepted light and a genetic term. This approach was used for predicting the behaviour of individual genotypes in virtual multi-genotype canopies. A large effect of competitiveness was observed in multi-genotype but not in single-genotype canopies, resulting in a bias for genotype comparisons in breeding fields.

## Teaser

A revised Monteith equation allows better dissection of environmental *vs* genetic controls of light interception and use efficiency in canopies and evaluation of bias associated with inter-genotypic competition and canopy heterogeneity.

## Introduction

Light interception is a main determinant of the genetic variability of biomass accumulation, together with photosynthetic rate (Zhu *et al.*, 2010; Reynolds *et al.*, 2012). It is defined and measured as the proportion of incident light that is intercepted by a canopy (radiation interception efficiency; RIE). It is essentially linked to leaf area, itself subjected to tight genetic and environmental controls (Tardieu *et al.*, 2014), but plant architecture also largely contributes to light interception and plant performance as suggested by the success of breeding programmes affecting leaf erectness (Mantilla-Perez and Salas Fernandez, 2017). Leaf photosynthetic rate is a second determinant of biomass accumulation. It is usually determined based on instantaneous measurements at the leaf level that need to be performed on several appropriate leaf samples per plant at different times of the day to represent the spatial and temporal variabilities of photosynthetic activity. This is nearly incompatible with high throughput measurements, thereby impeding genetic analyses. Hence, it is useful to consider a proxy of it in phenomic studies. Radiation use efficiency (RUE), defined as the dry canopy biomass produced per unit intercepted radiation, presents a strong genetic positive correlation with net photosynthetic rate at leaf level (Cabrera-Bosquet *et al.*, 2016). A major interest of RUE is that it can be estimated for thousands of plants in a phenotyping platform, whereas single leaf–atmosphere or canopy–atmosphere gas exchange measurements can hardly be managed at this spatial scale (Koester *et al.*, 2016; Moualeu-Ngangue *et al.*, 2017; Stinziano *et al.*, 2017). The relative contributions of RIE and RUE to biomass accumulation have been synthesized in a widely accepted model (Monteith 1977), used since then for physiological dissections of biomass accumulation (Louarn *et al.*, 2008; Chen *et al.*, 2015), for ecological studies (Onoda *et al.*, 2014), and for modelling at scales ranging from single leaf to regions, for example in analyses of climate change (Asseng *et al.*, 2013; Martre *et al.*, 2015). Both RIE and RUE show large genotypic variabilities (Maddonni *et al.*, 2001; Moreau *et al.*, 2012; Cabrera-Bosquet *et al.*, 2016), so it is now essential to analyse their genetic architectures in a range of environmental scenarios in order to relate them to biological pathways, as done in studies of other complex traits (e.g. flowering time in Buckler *et al.*, 2009; grain yield in Millet *et al.*, 2016) and to identify sources of genetic progress in a changing climate (Lobell *et al.*, 2011; Tardieu *et al.*, 2018).

The model proposed by Monteith (1977) analyses biomass accumulation by a crop canopy as a whole, so the contribution of individual plants can only be identified if the involved plants present similar RUEs and RIEs. This assumption is most often acceptable in large fields involving one genotype. However, there is an increasing interest in multi-genotype canopies for their resilience to diseases (Sapoukhina *et al.*, 2013) or risk minimization (Tilahun, 1995), or in association of bushes or small trees with crops for better use of resources over 1 year (Luedeling *et al.*, 2016). Furthermore, many phenotyping activities are carried out with multi-genotype canopies, for example in field experiments in the first steps of plant breeding or in most phenotyping platforms. In addition, many agronomical experiments or breeding programmes involve small plots separated by paths, thereby introducing heterogeneous situations among plants for light interception. Progress in phenotyping now allows precise estimation of the individual leaf area and spatial arrangement of plants in phenotyping platforms (Alvarez Prado *et al.*, 2018), or leaf area index and gap fraction in the field (Liu *et al.*, 2017), thereby potentially allowing calculation of RIE and RUE at the scale of micro-plots in the field or of plants in phenotyping platforms. A methodological challenge is now to take into account the non-homogeneity of canopies and the plant-to-plant interactions to identify the genetic and environmental contributions to RIE and RUE. Indeed, RIE and RUE are associated with environmental terms that considerably reduce their heritabilities and therefore the power of their genetic analyses. It is noteworthy that this is already the case for RUE in any canopy because of its dependence on photosynthetic photon flux density (PPFD) (Warren-Wilson *et al.*, 1992; Onoda *et al.*, 2014; Chen *et al.*, 2014*b*).

A difficulty for assessing the effect of canopy heterogeneities on light interception is the near-infinite number of cases involving a number of genotypes with any spatial arrangement (density, orientation, position of paths). Here we propose a two-step approach for dealing with multi-genotype canopies. First we characterize the capability for light interception of individual plants of each genotype in a phenotyping platform, and then analyse RIE and RUE of any virtual canopy that combines genotypes and spatial arrangements. High-throughput phenotyping platforms now allow automatic acquisition of multi-view images of the plants, which can be used to estimate leaf area and biomass of individual plants (Rajendran *et al.*, 2009; Hairmansis *et al.*, 2014; Knecht *et al.*, 2016; Tardieu *et al.*, 2017; Brichet *et al.*, 2017), and to reconstruct realistic 3D architecture of plants (Pound *et al.*, 2014, McCormick *et al.*, 2016). We have recently proposed an approach that combines a 2D modelling of local light intensity in a greenhouse with a stochastic 3D model of light interception, which allows one to estimate, for each individual plant, the incident light flux over the considered plant, the amount of intercepted light and RUE (Cabrera-Bosquet *et al.*, 2016). However, this method does not yet explicitly take plant-to-plant variations and inter-genotypic interactions into account.

We propose here a revision of the Monteith equation that dissects RIE and RUE into genetic and environmental components. It can be used in heterogeneous canopies in a phenotyping platform, and allows simulating light interception and biomass accumulation of multi-genotype, non-continuous canopies in the field. Using this method, we have first explored the genetic variabilities of the terms that underline RIE and RUE in 255 maize hybrids, resulting in an increase in heritability and quality of quantitative trait locus (QTL) detection, compared with the direct estimates of RIE and RUE by the Monteith model. We have then simulated virtual multi-genotype canopies to test the extent to which the canopy heterogeneity affects biomass production of individual genotypes.

## Materials and methods

### Plant material and experimental conditions

A maize hybrid population was generated by crossing a common flint parent (UH007) with 255 dent lines presenting a restricted flowering window. Details can be found in Millet *et al.* (2016). Three experiments were conducted, in winter 2013, spring 2013, and spring 2016, in the PhenoArch phenotyping platform (Cabrera-Bosquet *et al.*, 2016) hosted at the M3P, Montpellier Plant Phenotyping Platforms (https://www6.montpellier.inra.fr/lepse/M3P). Plants were grown in polyvinyl chloride (PVC) 9 litre pots (0.19 m diameter, 0.4 m high) filled with a 30:70 (v/v) mixture of a clay and organic compost. Soil water content in pots was maintained at target values by daily watering of each pot using watering stations made up of weighting terminals with 1 g accuracy (ST-Ex, Bizerba, Balingen, Germany) and high-precision pump-watering stations (520U, Watson Marlow, Wilmington, MA, USA). A randomized complete block design was used where each hybrid was replicated three times. Within the platform, air temperature and humidity were measured at six positions every 15 min (HMP45C, Vaisala Oy, Helsinki, Finland). Greenhouse temperature was maintained at 26 ± 3 °C during the light period and 18 ± 1 °C during the night. Supplemental light was provided during the daytime when external solar irradiation dropped below 300 W m^−2^ and during the night to extend the photoperiod with 400 W HPS Plantastar lamps (Osram, Munich, Germany) with 0.4 lamps m^−2^. The resulting daily photoperiod was 12 h. Time corrected for temperature (thermal time) was expressed in equivalent days at 20 °C (d_20°C_, Parent and Tardieu, 2012). Full details of experimental and environmental conditions are described in Alvarez Prado *et al.* (2018).

### Estimating plant leaf area, biomass and 3D architecture

Red–green–blue (RGB) images (2056 × 2454 pixels) of each plant were taken daily with 13 views (12 side views from 30° rotational difference and one top view) by using the imaging units of the platform. Each unit is composed of a cabin involving top and side RGB cameras (Grasshopper3, Point Grey Research, Richmond, BC, Canada) equipped with 12.5–75 mm TV zoom lens (Pentax, Ricoh Imaging, France) and LED illumination (5050–6500 K colour temperature). Images were captured while the plant was rotating at constant rate using a brushless motor (Rexroth, Germany) and were precise enough for picturing the cabin with negligible spherical aberration (<0.1%). Top and side cameras were calibrated using reference objects in order to convert pixels into mm^2^ and to estimate camera poses for 3D reconstruction. Plant pixels from each image were segmented from those of the background by using a set of threshold algorithms and morphological operators using OpenCV libraries (Bradski and Kaehler, 2008; http://opencv.org; see Brichet *et al.*, 2017 for details). They were used for estimating the whole plant leaf area and fresh biomass via calibration curves with real plants of different genotypes at different phenological stages (Supplementary Fig. S1 at *JXB* online). At the end of the experiment, plants were harvested and the above ground fresh and dry biomasses were recorded.

The 13 images taken for each plant at each date were used to reconstruct plant 3D architecture. The volume corresponding to each plant was computed using a space carving algorithm (Kutulakos and Seitz, 2000) and represented by a 3D set of voxels (0.512 cm^3^ per voxel). This voxel set was transformed into a triangular mesh by using the marching cube algorithm (Lorensen and Cline, 1987) implemented in the scikit-image package (Van der Walt *et al.*, 2014). A custom-made software pipeline, PHENOMENAL (version 1.3.0), processed the images, stored intermediate results, and distributed the computation on the grid via the cyber-infrastructure InfraPhenoGrid (Pradal *et al.*, 2017) embedded in the scientific workflow system OpenAlea (Pradal *et al.*, 2008, 2015).

### Estimating incident radiation and dissecting radiation interception efficiency

2D maps of light transmission through the greenhouse were built by using hemispherical images taken every square metre in the platform (Cabrera-Bosquet *et al.*, 2016). This allowed estimation of the amount of light received daily by each plant within the platform for every day of the year based on light transmission of direct and diffuse light and on incident PPFD measured above the platform roof every 15 min (SKS 1110, Skye Instruments, Llandrindod Wells, UK). This amount was summed to the PPFD emitted by lamps for calculation of PPFD per unit horizontal surface area corresponding to each plant within the platform (*R*_*i*_, mol m^−2^ d^−1^).

The maize canopy in the platform was reconstructed every day based on the 3D architecture of each plant and its position in the greenhouse. The daily integral of intercepted photon flux was calculated for each plant (*R*_plant_, mol plant^−1^ d^−1^) by using the RATP light model (Sinoquet *et al.*, 2001, version number 5955). The scattering coefficient of the RATP model (the sum of leaf transmittance and reflection) was assumed to be 16%, and the canopy clumping factor was set to 0.8. The reconstructed 3D canopy in the RATP model was placed within a rectangular regular grid, with grid cells length, width, and height equal to 0.2, 0.2, and 0.1 m, respectively. Each cell was characterized by the leaf area density (cm^2^ cm^−3^) obtained by summing the area of all 3D mesh triangles included in the cell, regardless of the plant they originated from. The leaf angle distribution of the canopy was calculated as the mean of angles of all triangles of the 3D mesh. The amount of light intercepted by each cell was calculated every day by dividing the daily photon flux integral above the greenhouse (mol m^−2^ d^−1^) into 46 beam angles using a sky radiance distribution model, and computing the beam extinction through the grid. The amount of light intercepted by each plant, *R*_plant_, was obtained by summing the radiation intercepted by each cell occupied by the plant, weighted by the relative contribution of the considered plant to the cell area and multiplied by the light transmission at the corresponding *x*–*y* position in the platform (Cabrera-Bosquet *et al.*, 2016).

### Dissecting radiation use efficiency

RUE (g fresh weight mol^−1^ photons) of each plant was estimated as the slope of linear regression between above-ground biomass production and cumulative intercepted radiation of the plant in the interval 30–50 d_20°C_ (Cabrera-Bosquet *et al.*, 2016).

Physiologically, RUE is not a genotypic constant but a variable dependent on the amount of intercepted light per unit leaf area (*R*_leaf_; µmol m^−2^ s^−1^). Here, the daily average of *R*_leaf_ was calculated by first converting the unit of *R*_plant_ (mol plant^−1^ d^−1^) into µmol plant^−1^ s^−1^ based on the photoperiod of 12 h, then dividing *R*_plant_ by the leaf area (LA) of the day (*R*_leaf_=*R*_plant_/LA). Due to the strong non-linear relationship between RUE and *R*_leaf_ (Warren-Wilson *et al.*, 1992; Chen *et al.*, 2014*a*), we linearized RUE using a log transformation in order to facilitate further analyses:

$$\text{lnRUE}=aR_{\text{leaf}}+b+\varepsilon_{\text{RUE}}$$ (1)

where *a* and *b* are the slope and intercept of the linear regression, respectively, and ε_RUE_ is the residual of the regression. Therefore, the term RUE in the Monteith equation was replaced by:

$$\text{RUE}=e^{\varepsilon \text{RUE}}\times e^{\left(a\times R_{\text{leaf}}+b\right)}$$ (2)

### Simulating biomass accumulation in virtual canopies

The 3D plant architecture corresponding to each plant was used to construct virtual fields with different canopy configurations (i.e. mixture of different genotypes, variations in plant density, and different developmental stages of the plants). Each genotype was represented in virtual canopies with random replications of the three plants observed in the experiment of spring 2016. In total, more than 10 000 simulations were managed by an extended version of the cyber-infrastructure InfraPhenoGrid (Pradal *et al.*, 2017, version PHENOARCH 0.7). The amount of light intercepted by each individual plant in the virtual fields (*R*_plant_) was calculated every day by using the RATP light model. Daily biomass accumulation of individual plants (g fresh weight plant^−1^ d^−1^) was calculated in the virtual canopies with the genotypic values of *R*_plant_, leaf area and ε_RUE_.

### Genome-wide association analysis

The 255 lines were genotyped using a 50K Infinium HD Illumina array (Ganal *et al.*, 2011), a 600K Axiom Affymetrix array (Unterseer *et al.*, 2014), and a set of 500K markers obtained by Genotyping by Sequencing (GBS; S. Negro, S. Nicolas and A. Charcosset, personal communication). After data quality control, 758 863 polymorphic single nucleotide polymorphisms (SNPs) were retained for the analysis. For each single experiment, a genome-wide association analysis (GWAS) was performed, with the methods presented in Millet *et al.* (2016), for the original components of the Monteith equation (biomass, RIE, and RUE), and for the components of our new dissection. We used a single locus mixed model:

Y=μ+Xβ+G+E (3)

where *Y* is the vector of phenotypic values, μ the overall mean, *X* the vector of SNP scores with additive effect β, *G* the random polygenic effects, and *E* the residual effects (see Millet *et al.*, 2016 for details on the method and kinship matrix calculation). An initial set of significant SNPs was selected, including all SNPs with –log_10_*P*-value larger than 5. Candidate SNPs distant less than 0.1 cM were considered as belonging to a common QTL, described via the most significant SNP in the QTL. Broad-sense heritability (*H*^2^) was estimated by *σ*_G_^2^/(*σ*_G_^2^+*σ*_e_^2^/*r*), where *σ*_G_^2^, *σ*_e_^2^, and *r* are genotypic variance, residual variance and number of plant replicates per genotype.

## Results

### Introducing an environmental term that accounts for the effect of spatial heterogeneities, thereby increasing the heritability of radiation interception efficiency

An environmental term is justified because spatial heterogeneities in the field, such as paths, borders and discontinuities in the canopy, considerably affect light interception by individual plants. In phenotyping platforms, border plants receive more light than those inside the canopy. If not corrected in the model, these heterogeneities may considerably decrease the heritability of RIE in genetic analyses.

We have first defined radiation interception efficiency at the plant level (RIE_plant_) as the ratio of the amount of light intercepted by a plant to the amount of incident light summed over a spatial domain centred on the plant and whose area equals the space allocated to each plant, i.e. the reciprocal of plant density.

$${\text{RIE}}_{\text{plant},i}=R_{\text{plant},i}\times D/R_{i}$$ (4)

where *R*_plant,*i*_ (mol plant^−1^ d^−1^) is the total light intercepted by plant *i* from the different directions of incidence (as calculated by the light model), *R*_*i*_ (mol m^−2^ d^−1^) is the photon flux density through a horizontal surface above the plant, and *D* is plant density (plants m^−2^ soil surface area). Hence, RIE_plant,*i*_ is unitless (as RIE is in the Monteith model).

**Fig. 1.**
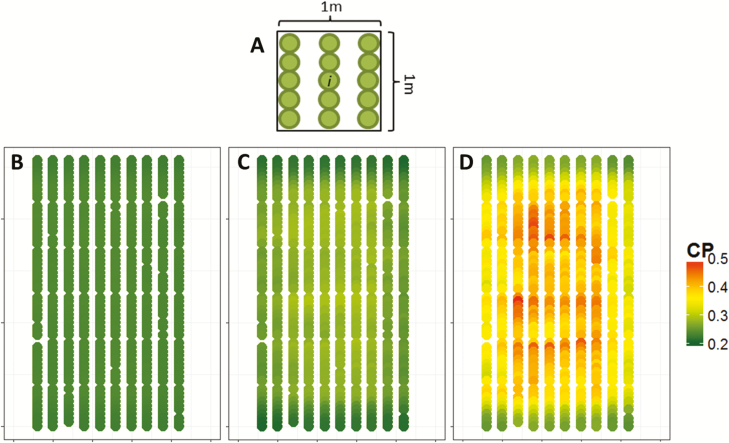
Map of the canopy competition pressure (CP, m^2^ leaf area per plant) in the phenotyping platform during the experiment of spring 2016. (A) A schematic representation of the moving window centred on plant *i* for each plant of the canopy. (B–D) Thermal times at the beginning (B), middle (C), and end (D) of the experiment were 14.5, 31.2, and 45.0 equivalent days at 20 °C, d_20°C_, after sowing, respectively.

**Fig. 2.**
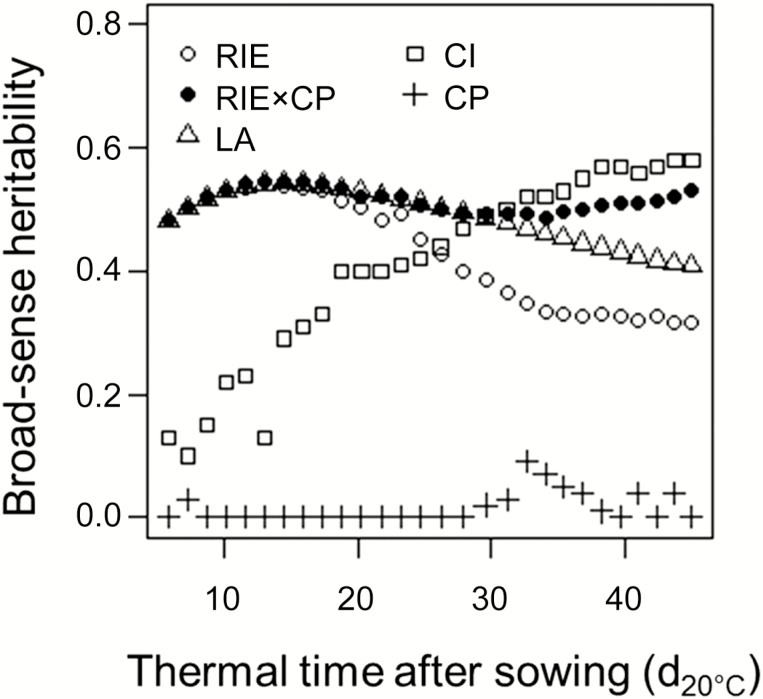
Broad-sense heritabilities of radiation interception efficiency at plant level (RIE_plant_, unitless, open circles), of RIE_plant_ corrected for local competition pressure (RIE_plant,CP_, m^2^ leaf area per plant, black circles), of plant competitiveness index (CI, unitless, open squares) and of canopy competition pressure (CP, m^2^ leaf area per plant). The heritability of leaf area per plant (LA, m^2^ plant^−1^, open triangles) is also presented for comparison with other indices. Data from the experiment of spring 2016.

We then introduced a coefficient, the canopy competition pressure (CP, m^2^ leaf area per plant), which takes spatial heterogeneities into account via the reciprocal of light interception per unit leaf area in the neighbourhood of the considered plant. It is defined as the ratio of local mean leaf area (LA_c,*i*_, m^2^ leaf area per plant) to the mean radiation interception efficiency in the same spatial window. In the experiments considered here, this area hosted 15 plants (Fig. 1A).

$${\text{CP}}_{i}={\text{LA}}_{\text{c},i}/{\text{RIE}}_{\text{c},i}$$ (5)

where RIE_c,*i*_ is the mean RIE_plant_ in this window (unitless). CP has lower values in border plants than within the canopy because RIE_c,*i*_ is more affected than mean leaf area by the presence of neighbouring plants. This can be visualized in Fig. 1B–D, which represents the spatial distribution of CP in the greenhouse at three dates. At the beginning of the plant cycle [Fig. 1B; at 14.5 equivalent days at 20 °C (d_20°C_) after sowing], CP was uniform in the greenhouse, meaning that plants inside the canopy were too small to cause mutual shading. A spatial pattern began to appear 31.2 d_20°C_ after sowing and was clear at 45 d_20°C_. At these dates, the CP of border plants was similar to that calculated for the first date, whereas plants within the canopy presented much higher CP (for day-to-day changes in CP every day; see Supplementary Video S1). The non-uniform CP inside the canopy reflects the fact that the relationship between LA and RIE is not linear, so the ratio tends to increase for higher leaf area in the considered window. The heritability of CP was close to zero throughout the experimental period (Fig. 2), suggesting that CP is an essentially environmental variable. CP was also independent of leaf area (*r*^2^=0.08).

Why correcting RIE_plant_ for the effect of CP is of interest can be visualized in Fig. 2. Indeed, the broad-sense heritability of the raw value of RIE_plant_ decreased rapidly from 20 to 30 d_20°C_ after sowing due to the border effect that reduced the repeatability of RIE_plant_ for a given genotype. The effect of CP on RIE_plant_ was negligible at the beginning of the experiment but largely increased with time. The heritability of RIE_plant_ corrected for CP (RIE_plant,CP_, defined as the product of both terms) did not differ from that of RIE_plant_ at the beginning of the experiment, but remained at nearly constant values throughout the experiment because the environmental component of RIE_plant_ was removed (Fig. 2). For example, correcting RIE_plant_ by CP increased heritability from 0.32 to 0.53 at the end of the experiment (Fig. 2).

### Introducing a competitiveness term that accounts for inter-genotypic interactions in light interception

RIE_plant,*i*_ is similar in all plants of a homogeneous canopy because the amount of light harvested by plant *i* outside its spatial domain matches the amount of light harvested by the leaves of neighbouring plants inside its domain. This is not the case in a heterogeneous canopy because both amounts may appreciably differ, so RIE_plant,*i*_ can largely vary between individual plants and can be greater than unity for a plant that is more competitive than its neighbours.

The above-mentioned effect was taken into account via a plant competitiveness index (CI) defined as the ratio of radiation interception efficiency of plant *i* normalized by its leaf area (RIE_plant,*i*_/LA_*i*_), to the radiation interception efficiency in the spatial window centred on plant *i* (Fig. 1A) corrected by its mean leaf area (RIE_c,*i*_/LA_c,*i*_). CI is therefore unitless. It equals 1 if plant *i* intercepts the same amount of light per leaf area as the average of its neighbours, and is higher if it intercepts more than its neighbours.

$${\text{CI}}_{i}=\left({\text{RIE}}_{\text{plant},i}/{\text{LA}}_{i}\right)/\left({\text{RIE}}_{\text{c},i}/{\text{LAI}}_{\text{c},i}\right)$$ (6)

where LA_*i*_ is the leaf area of plant *i.* Other variables are defined as in Eqs (4) and (5).

**Fig. 3.**
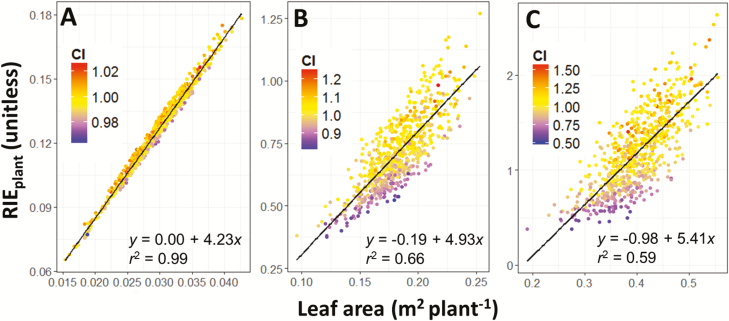
Relationship between radiation interception efficiency by individual plants (RIE_plant_, unitless) and leaf area (m^2^ plant^−1^) at the beginning (A), middle (B), and end (C) of the experiment of spring 2016. The colours of circles denote the value of the competitiveness index (CI, unitless) for each plant. Note that plants with low CI (purple) are below the regression line. Thermal times after sowing are 14.5 d_20°C_ (A), 31.2 d_20°C_ (B), and 45.0 d_20°C_ (C) for the experiment of spring 2016.

**Fig. 4.**
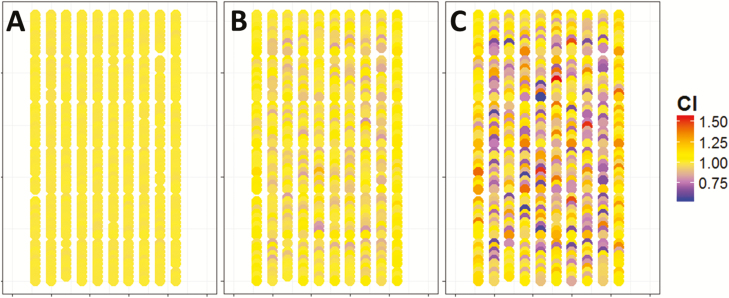
Map of the competitiveness index (CI, unitless) at the beginning (A), middle (B), and end (C) of the experiment of spring 2016. Each point represents a plant in the platform, placed at the *x–y* coordinates of plants. The colours of circles denote the value of CI. Thermal time at the beginning (A), middle (B), and end (C) of the experiment were 14.5, 31.2, and 45.0 d_20°C_, respectively.

At the beginning of the plant cycle (14.5 d_20°C_ after sowing), CI was close to unity because plants had little interactions with their neighbours, so RIE_plant_ was closely related to leaf area (Figs 3A, 4A). From the middle of the experiment onwards, CI became more diverse between plants (31.2 d_20°C_, ±20%; Figs 3B, 4B) and ranged from 0.6 to 1.4 at 45 d_20°C_ (Figs 3C, 4C). At this time, RIE_plant_ was related to plant leaf area, but with a large variability around the regression line (up to ±40%). Whether data points were located below or above the regression line depended on the value of CI of the considered plant (Fig. 3C; for a temporal dynamic of CI, see Supplementary Video S2). Hence, RIE_plant_ could be appreciably higher than 1 for plants having high CI (Fig. 3B, C).

**Fig. 5.**
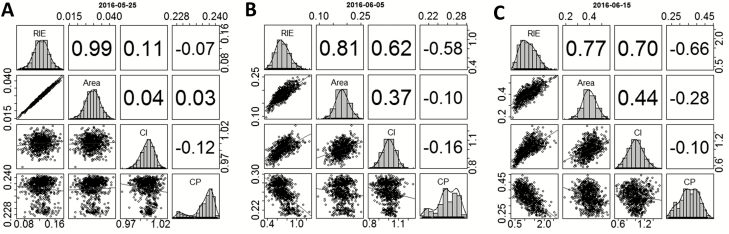
Correlations between RIE_plant_ (unitless), leaf area (m^2^ plant^−1^), competitiveness index (CI, unitless), and competition pressure (CP, m^2^ leaf area per plant) at the beginning (A), middle (B), and end (C) of the experiment of spring 2016. Pearson correlation coefficients are shown in the upper panel. Thermal time at the beginning, middle, and end of the experiment was 14.5, 31.2, and 45.0 d_20°C_, respectively. Daily changes of these correlations throughout the whole experiment can be found in Supplementary Video S3.

The heritability of CI increased with time, indicating that the genetic control of CI became appreciable after canopy closure (Fig. 2). CI only explained 0.01% of the variation of RIE_plant_ at the beginning of the plant cycle in the experiment of spring 2016, (14.5 d_20°C_; Fig. 5A), with a heritability of 0.13. It explained 49% of the variations of RIE_plant_ later on (Fig. 5C; Supplementary Video S3), with a heritability of 0.58. The heritability of CI was also high at the end of the experiments of winter and spring 2013 (0.67 and 0.68, respectively; Supplementary Table S1).

### Dissecting radiation use efficiency into genetic and environmental components

**Fig. 6.**
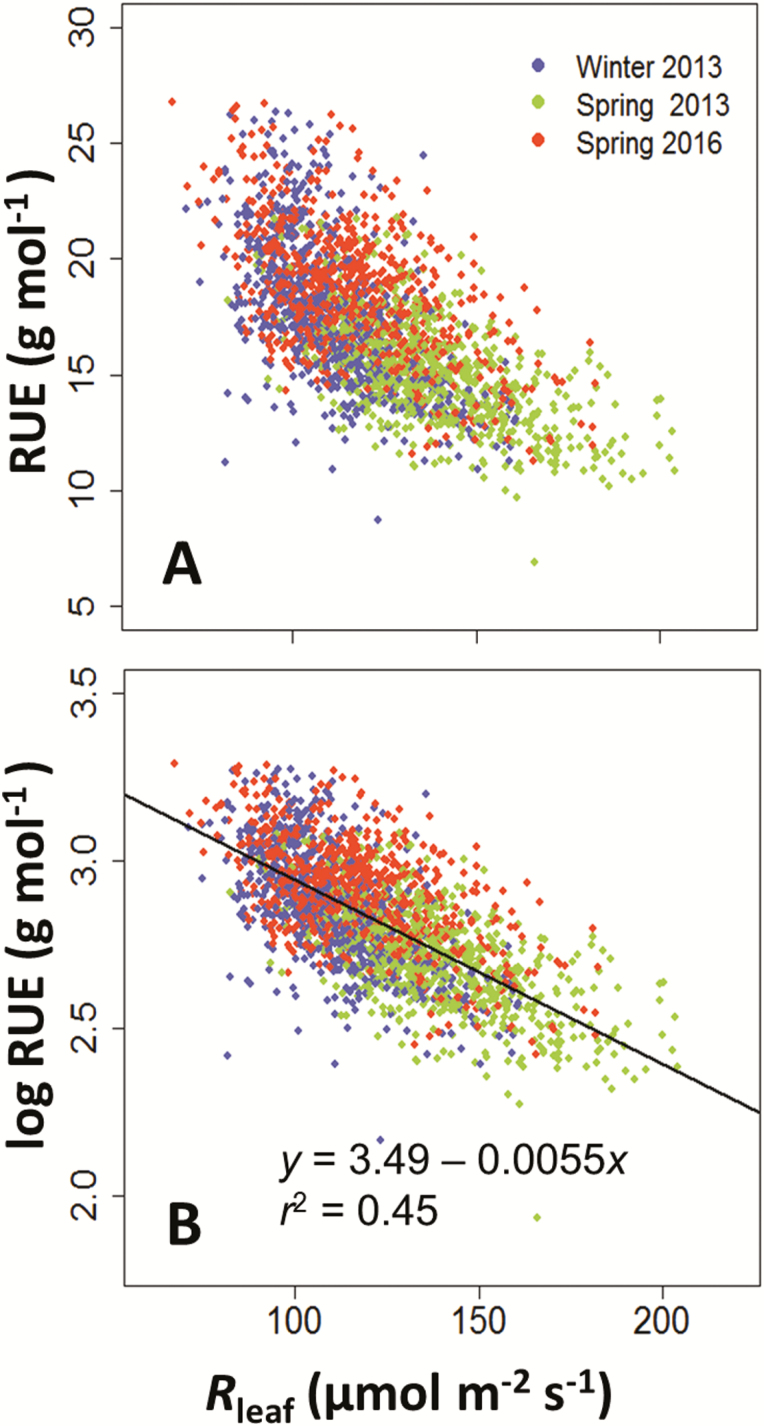
Radiation use efficiency (RUE) as a function of light intercepted per unit leaf area (*R*_leaf_), with either raw (A) or log-transformed (B) values.

**Fig. 7.**
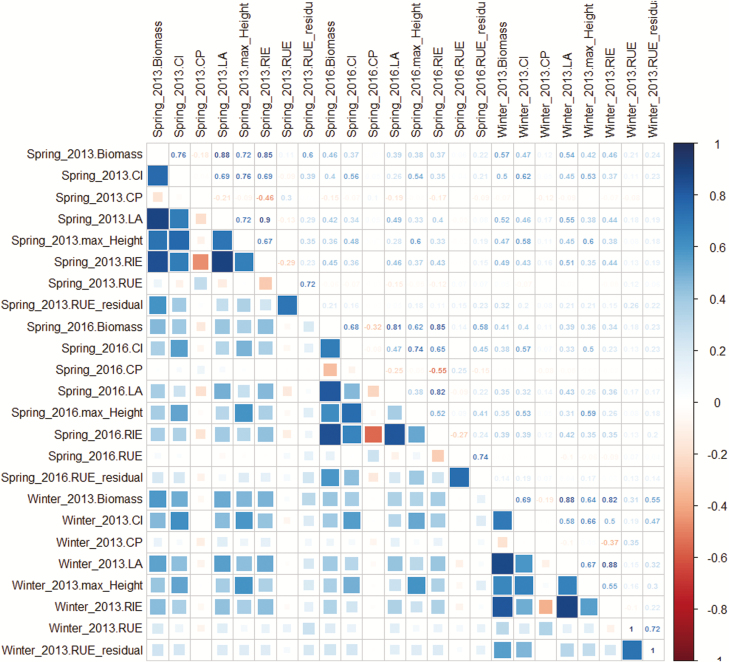
Correlations between genotypic means of studied traits. Pearson correlation coefficients are shown in the upper right part of the figure. Biomass: biomass at the end of the experiment; CI, CP, and LA: plant competitiveness index, canopy competition pressure, and leaf area, respectively, at the end of the experiment; max_Height: maximal plant height of the experiment; RIE: mean RIE_plant_ throughout the experiment; RUE: mean radiation use efficiency; RUE_residual: residual from the linear regression in Fig. 6B.

Raw values of RUE varied 3-fold, from 9 to 25 g FW mol^−1^ between plants, with a low heritability (Table 1). They showed a strong dependency on the mean intercepted light per unit leaf area of each plant [*R*_leaf_ in Eq. (1); Fig. 6A]. Hence, we have dissected RUE into an environmental term, intercepted light, and a genetic term estimated as the mean value of the residuals corresponding to a genotype in the regression between RUE and *R*_leaf_, after linearization via a logarithmic transformation [Eq. (1); Fig. 6B; *r*^2^=0.45]. The genotypic differences in RUE were therefore characterized by this residual term, named ε_RUE_ [Eq. (5)], which can be either positive or negative depending on the genetic value of the residual in relation to the regression line that was common to all genotypes. ε_RUE_ had higher heritability than raw values of RUE (Table 1), and higher correlation with the genotypic values of biomass (Pearson correlation coefficient of 0.55–0.60 for ε_RUE_*vs* 0.11–0.31 for RUE; Fig. 7).

**Table 1.** Broad-sense heritability (*H*^2^), minimum (Min) and maximum (Max) genotypic values, and number of quantitative trait loci (QTLs) for traits in this study

	*H* ^2^	Min	Max	QTLs	Colocalization of QTLs
Original variables in Monteith equation/uncorrected traits					
RIE	0.42	0.29	0.81	6	Biomass (4), CI (1), RIE_plant_ (3), RIE_plant,CP_ (4)
RUE	0.17	12.02	24.78	6	
RIE_plant_	0.32	0.53	1.93	8	Biomass (4), CI (2), RIE (3), RIE_plant,CP_ (4),
Indices proposed in Eqs (5)–(7)					
CP	0.04	0.25	0.46	0	None
CI	0.58	0.60	1.47	7	Biomass (1), RIE (1), RIE_plant_ (2), RIE_plant,CP_ (3)
ε_RUE_	0.42	−0.08	0.11	4	None
RIE_plant,CP_	0.53	0.20	0.67	15	Biomass (4), leaf area (1), CI (3), RIE (4), RIE_plant_ (4)

Uncorrected traits stand for original traits in the Monteith equation: radiation interception efficiency (RIE, unitless), radiation use efficiency (RUE, g fresh biomass mol^−1^ photon) and RIE at the end of the experiments (RIE_plant_, unitless). The traits proposed in this study are canopy competition pressure (CP, m^2^ leaf area per plant), plant competitiveness index (unitless), and RIE_plant_ corrected for local variations of CP (RIE_plant,cp_). Colocalizations between traits are also presented (number of QTL colocalizations shown in parentheses). Data refer to the experiment of spring 2016. Values corresponding to other traits (e.g. leaf area or plant height) can be found in Supplementary Tables S1, S2.

### The resulting expression of the Monteith equation improved the genetic analysis of biomass accumulation

Based on the results presented above, we propose a modification of the Monteith equation for a multi-genotype canopy, which allows identifying genotype-dependent variables with high heritability on one hand and environmental terms on the other hand:

$$\Delta \text{BM}/\Delta t=R_{i}\times {\text{RIE}}_{\text{plant}}\times \text{RUE}/D=R_{i}\times \left(\text{LA}\times \text{CI}/\text{CP}\right)\times \left(e^{\varepsilon \text{RUE}}\times e^{\left(a\times R_{\text{leaf}}+b\right)}\right)/D$$ (7)

where ΔBM/Δ*t* is the increase in biomass production per simulation step, *R*_*i*_ and CP are environmental variables, *a* and *b* are statistical parameters valid for the whole panel of hybrids and LA, CI, and ε_RUE_ are genotype-dependent. *R*_leaf_ is the ratio of *R*_plant_ to LA, and so does not represent an additional variable.

A first application of Eq. (7) was to quantify the relative contributions of the genetic variations in LA, CI and ε_RUE_. For instance, the genetic variations in ε_RUE_ ranged between −0.126 and 0.109 in all experiments, resulting in an influence on biomass up to ±12%, much less than the genetic variations in LA and in CI (Table 1; Supplementary Table S1).

We have run GWAS analyses of the terms in Eq. (7) to test the extent to which the above analysis allowed better genetic dissection of biomass accumulation, and to identify which variables share part of their genetic controls.

1. The identification of the role of an environmental term, CP, improved the heritability of RIE_plant_ (Fig. 2), thereby allowing identification of more QTLs (15 and eight QTLs, respectively, after and before correction; Table 1). As expected for an environmental term, the heritability of CP was close to 0 and no QTL was identified.
2. The genotypic dependency of CI after canopy closure, shown by the increase with time of heritability (Fig. 2), resulted in seven QTLs, three of them co-localizing with QTLs of RIE corrected by CP (on bin 3.02, 4.09 and 5.04 with consistent allelic effects; Table 1; Supplementary Table S2), and one of them co-localized with biomass and RIE with consistent negative allelic effects (bin 4.09, 234.7 Mb; Supplementary Table S2). This indicates that the dissection proposed here improved the genetic dissection of biomass.
3. The dissection of RUE into an environmental term and a genetic term also improved genetic analysis. Indeed, 10 QTLs were detected for ε_RUE_, one of them co-locating with a QTL for biomass with consistent allelic effects (bin 1.10, 275.1 Mb; Supplementary Table S2).

This analysis also allowed identification of variables to which CI was genetically linked. CI was related to plant height (*r*^2^=0.55), with taller plants being more competitive than their neighbours (Fig. 7; Supplementary Fig. S2). Conversely, CI was essentially independent of leaf area (*r*^2^=0.00–0.19; Fig. 5; Supplementary Video S3). There was only one co-location between QTLs of leaf area and of CI (bin 7.02), but this QTL also involved plant height, thereby conferring a higher competitiveness to plants carrying this allele (Supplementary Table S2). CI was also unrelated to CP (*r*^2^= 0.01; Fig. 5; Supplementary Video S3).

### The modified Monteith equation also allowed estimation of the bias associated with heterogeneities of competitiveness in typical breeders’ designs

**Fig. 8.**
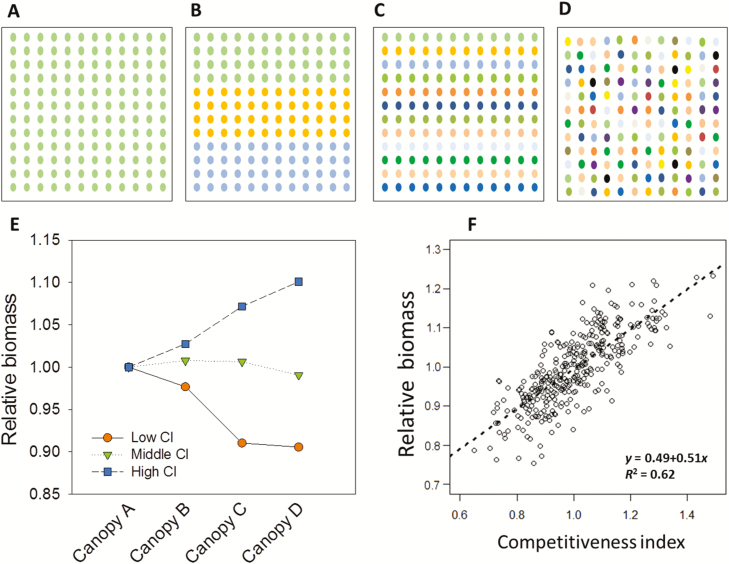
Virtual experiments simulating biomass of different genotypes under different canopy complexities: (A) canopy A, a simple, large plot canopy consisting of one genotype; (B) canopy B, a canopy with four rows per genotype for nine genotypes (only three presented here); (C) canopy C, a canopy with one row per genotype for nine genotypes; (D) canopy D_all_, a canopy with random mixture of all genotypes. (E) Ratio of the simulated biomass in canopies A–D for a given genotype to that in a mono-genotype canopy (relative biomass). Colours denote the group of genotypes with low (orange circles, three genotypes), middle (green inverted triangles, three genotypes) and high (blue squares, three genotypes) values of competitiveness index (CI). (F) Relationship between the ratios between biomasses of canopies D and A calculated for 255 genotypes, and the plant competitiveness index of the same genotypes as measured in the phenotyping platform.

Another interest of the modified Monteith equation [Eq. (7)] is that it can assess the effects of heterogeneities on biomass accumulation by either whole canopies or individual plants in the canopy. In particular, we have used Eq. (7) and data obtained from platform experiments to assess the bias associated to plant distribution in typical canopy structures used in breeding programmes. For that, five typical canopies with increasing complexity were generated: (i) large plot single-genotype canopies, simulated for all 255 genotypes, resembling those in farmers’ fields (canopy A, Fig. 8A); (ii) micro-plots of four rows harbouring one genotype each, typically used in experiments for genetic analyses [canopy B (Fig. 8B) simulated here for nine genotypes]; (iii) micro-plots with one genotype per row, a design corresponding to early generations of breeding programmes (canopy C, Fig. 8C); and (iv) a field with either nine or 255 genotypes (canopies D and D_all_, respectively; Fig. 8D showing canopy D_all_) distributed randomly, resembling a first generation breeding field, the experimental design of a phenotyping platform or a farmer’s field with mixed varieties for pest avoidance. The nine genotypes used in canopies B, C, and D were classified into three groups by their genotypic values of CI in the platform, namely low, middle, and high CI (0.73 ± 0.07, 0.99 ± 0.04, and 1.27 ± 0.06, respectively). The daily biomass production of each genotype in different canopies was then calculated based on Eq. (7) by using the genotypic values measured in the platform experiment.

Genotypes with high CI were favoured in multi-genotype canopies associated with breeding programmes (canopies B, C, and D) compared with their behaviour in pure canopies (canopy A), whereas genotypes with low CI performed better in canopy A than in canopies B, C, and D (Fig. 8E). This was in spite of the fact that genotypes performed similarly when simulated individually in large plots (3.58–3.79 kg m^−2^) and that the overall performance of canopy D was 3.63 kg m^−2^, similar to the mean performance of the same genotypes in large plots.

Hence, the inter-genotypic interactions in light capture resulted in an appreciable bias in the comparison of genotypes in multi-genotype canopies (from 3.08 to 4.21 kg m^−2^ in canopy D). The difference between simulated biomass between canopies A and D_all_ reached ±20% and CI explained more than 60% of these differences (Fig. 8F). Genotypes with intermediate CI performed the same in all canopies whereas the competitive advantage of plants with high CI increased with the degree of canopy heterogeneity, from canopy B to canopy D (Fig. 8E).

## Discussion

### The revised Monteith equation allowed better dissection of the genetic controls of biomass production

Comparing the performance of genotypes in multi-genotypic and heterogeneous canopies involves a risk of confusion of effects due to the structure of the canopy. Such canopies are used for many purposes, such as selection of the F2 to F4 generations of breeding, studies in phenotyping platforms (Cabrera-Bosquet *et al.*, 2016), minimizing risk in yield loss (Tilahun, 1995), maintaining productivity in subsistence agriculture (Smithson and Lenné, 1996) and increasing diversity for pest management in organic farming (Sapoukhina *et al.*, 2013).

Starting from the original Monteith equation (Monteith, 1977), our approach (Eq 7) quantitatively dissected biomass of individual plants in a multi-genotype canopy into environmental and genetic terms. By keeping the equation multiplicative as in the original Monteith equation, the revised equation provides a quantitative assessment of the variabilities of the genetic terms (Table 1) and of their impacts on genotypic biomass performance in heterogeneous canopies. The fact that the new genetic terms (LA, CI, and ε_RUE_) are more heritable (Table 1) and more reproducible between experiments than RIE and RUE (Fig. 7) indicates that the attempt to isolate environmental from genetic terms was at least partly successful. These genetic terms correlate better to biomass than to each other (Fig. 7) and their QTLs associate independently with biomass, thereby improving the ability to explore their genetic control (Tardieu and Tuberosa, 2010). A trade-off of this approach is that fresh biomass was taken into account, rather than dry biomass in the Monteith equation. Biomass estimation via imaging is based on plant volume. A mean correction term transforming fresh into dry biomass could have been used, but this would hide the potential genetic variability of plant water content, so we preferred keeping fresh biomass in the analysis.

### Genetic variations of expansive growth and RUE and their effects on canopy biomass production

Improving canopy photosynthesis or RUE has been proposed to be the next target for increasing crop yield (Zhu *et al.*, 2010; Ort *et al.*, 2015; Wu *et al.*, 2016), but our data suggest that the explored genetic variability of RUE, estimated via the term *e*^εRUE^in Eq. (7), contributed only up to ±12% of the variations in biomass production in the studied genotypes, much less than that of light interception (Supplementary Table S1). This finding is in agreement with virtual field experiments where the final leaf area explains 80% of the simulated biomass in simple canopies involving one genotype. The genetic variability of expansive growth therefore contributes more to biomass accumulation than that of photosynthesis (Fatichi *et al.*, 2014; Tardieu *et al.*, 2014). During the vegetative phase, rapid expansive growth not only increases source strength because of the deterministic relations between canopy leaf area, light interception and the size of photosynthetic organs (Chen *et al.*, 2015), but also sink strength via its indirect effect on silk growth (Turc *et al.*, 2016) that reduces the risk of abortion (Oury, *et al.*, 2016).

Our data also suggest that the reported 2-fold variations of RUE in maize in the field (Louarn *et al.*, 2008; Cicchino *et al.*, 2010; Wu *et al.*, 2016) or in a phenotyping platform (Cabrera-Bosquet *et al.*, 2016), might partly be a consequence of variations in light conditions (Fig. 6A). Between European sites, incident light during the same phenological stage in maize can vary 3-fold (Millet *et al.*, 2016). Meta-analysis has also shown that shade increases RUE by up to 200% when the availability of light reduces by more than 50% (Slattery *et al.*, 2013), fairly in agreement with our data (Fig. 6A).

Instead of fitting the parameters *a* and *b* in Eq. (7) for the whole maize panel, we also tried several apparently more deterministic approaches; for example, we considered *a* and *b* as genetic parameters characterizing the dependency of RUE to *R*_leaf_, fitted for each genotype (RUE=*e*^(*a*×*R*leaf+*b*)^). Coefficient *a* describes the decrease in RUE with the increasing light level. Therefore, it is related to the convexity factor and the maximal photosynthetic rate of the photosynthetic light response curves. Coefficient *b* can be interpreted as the initial slope of the photosynthetic light response curves. In this case, *a* and *b* could be biologically related to photosynthetic quantum yield and maximal photosynthetic capacity, respectively. However, we finally did not adopt this approach because of the strong correlation (*r*^2^=0.96) between genotypic values of *a* and *b*, together with the results of GWAS detecting no QTL for them. This suggests that apparent genetic differences in *a* and *b* were actually artefacts from the linear regression. We have therefore used a parsimonious alternative with one variable only, ε_RUE_, to represent the genetic term in RUE. Although the experimental errors are inevitably included in ε_RUE_, the latter variable is still more related to genetic controls of biomass than RUE, and presents a higher heritability (Fig. 7; Table 1).

### The competitiveness index helps assessing the bias due to inter-genotypic interactions in early breeding programmes

Our results suggest that heterogeneity in canopy structure may have appreciable implications for interpreting results from breeders’ early generations of selection and from phenotyping platforms. A recent experimental study also suggests large differences between yield of wheat in mixed and monoculture due to inter-genotypic competition (Weiner *et al.*, 2017). A consequence of inter-genotypic competition, also called competitive response (Bartelheimer, *et al.*, 2015), is that genotypes with good performance in one-genotype canopies might be discarded during the breeding process due to a low competitiveness compared with other genotypes. In the early generations of selection, breeders might therefore select genotypes with architectural advantages in competing light (high CI), such as plastic responses for shade avoidance, to increase competitive effects on the neighbouring plants (Abley *et al.*, 2016, Subrahmaniam *et al.*, 2018), especially under high planting density (Schmitt *et al.*, 1995, Weiner *et al.*, 2017), rather than selecting genotypes with high potential in simple canopies with one genotype.

## Supplementary data

Supplementary data are available at *JXB* online.

Fig. S1. Measured and predicted fresh biomass and leaf area.

Fig. S2. Relationship between competitiveness index and plant height at the end of the experiment of spring 2016.

Table S1. Broad-sense heritability (*H*^2^), minimum, maximum, and mean genotypic values of parameters obtained from the three experiment.

Table S2. QTLs for traits detected in the three experiments.

Video S1. Daily changes in competition pressure for each plant growing in the phenotyping platform experiment.

Video S2. Daily changes in competition index for each plant growing in the phenotyping platform experiment.

Video S3. Daily changes of correlations between RIE_plant_, leaf area, competitiveness index, and competition pressure throughout the experiment of spring 2016.

Supplementary Video S1

Supplementary Video S2

Supplementary Video S3

Supplementary Figures S1-S2 and Tables S1-S2

## Abbreviations

ε_RUE_: residual of the linear relationship between lnRUE and *R*_leaf_
CI: competitiveness index (unitless)
CP: competition pressure (m^2^, leaf area per plant)
D: plant density (m^−2^, plant per soil surface area)
d_20°C_: unit of time representing equivalent days at 20 °C (d)
*H*^2^: broad-sense heritability (unitless)
LA_c_: mean leaf area of a canopy (m^2^, leaf area per plant)
LA_*i*_: leaf area of a single plant *i* in the platform (m^2^ plant^−1^)
PPFD: photosynthetic photon flux density (mol m^−2^ d^−1^)
*R*_*i*_: daily integral of PPFD above the canopy of plant *i* in the platform (mol m^−2^ d^−1^)
*R*_leaf_: intercepted light per unit leaf area (µmol m^−2^ s^−1^)
RIE_c_: radiation interception efficiency at the canopy level (unitless);
RIE_plant_: radiation interception efficiency at the plant level (unitless)
RIE_plant,CP_: RIEplant corrected by CP (m^2^, leaf area per plant);
*R*_plant_: daily integral of intercepted PPFD of a single plant in the platform (mol plant^−1^ d^−1^)
RUE: radiation use efficiency (g mol^−1^, fresh weight per photons)
