# Supplementary material for: Genetic and environmental dissection of biomass accumulation in multi-genotype maize canopies
Source: J Exp Bot. 2019 Apr 18;70(9):2523–34. doi: 10.1093/jxb/ery309 (PMC6487589; doi:10.1093/jxb/ery309)
Supplement: Supplementary Figures S1-S2 and Tables S1-S2 [file ery309_suppl_supplementary_figures-s1-s2_tables-s1-s2.docx]

**Supplementary Information**

RESEARCH PAPER

**Genetic and environmental dissection of biomass accumulation in multi-genotypes maize canopies**

Tsu-Wei Chen^1*^, Llorenç Cabrera-Bosquet^1^, Santiago Alvarez Prado^1^, Raphaël Perez^1^, Simon Artzet^1,2^, Christophe Pradal^3^, Aude Coupel-Ledru^1^, Christian Fournier^1,2^, François Tardieu^1^

^1^INRA, UMR LEPSE, Montpellier, France; ^2^Virtual Plants, INRIA, CIRAD, INRA, 34095 Montpellier France; ^3^CIRAD, UMR AGAP, Montpellier, France

^*^Current address: Institute of Horticultural Production Systems, Leibniz Universität Hannover, Hannover, Germany [chen@gem.uni-hannover.de](mailto:chen@gem.uni-hannover.de)

Corresponding author

Tsu-Wei Chen: [chen@gem.uni-hannover.de](mailto:chen@gem.uni-hannover.de) ; Francois Tardieu francois.tardieu@inra.fr

Telephone: +49 511 762-19269

Fax: +49 511 762-3606


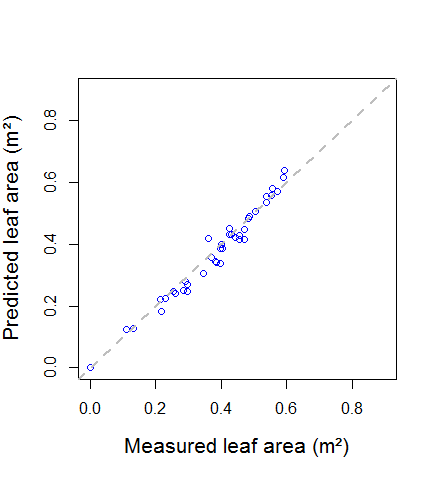

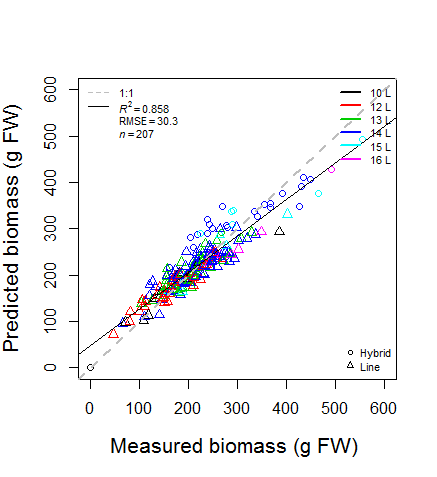


Figure S1: Relationships between measured and predicted whole plant fresh biomass and leaf area of different genotypes at different phenological stages.


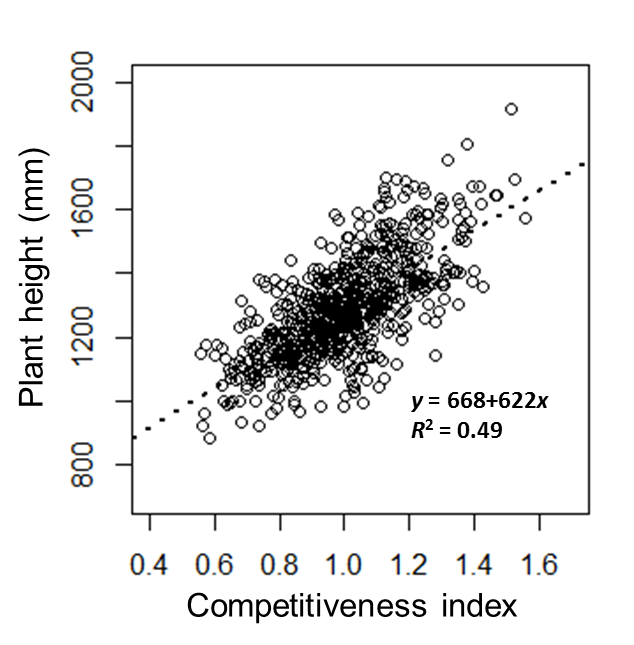


Figure S2: Relationship between competitiveness index (unitless) and plant height at the end of Exp. spring 2016.

**Table S1.** Broad-sense heritability (*H*^2^), minimum, maximum and mean genotypic values of measured fresh biomass (g plant^-1^), mean radiation interception efficiency (RIE, unitless), radiation use efficiency (RUE, g fresh biomass mol^-1^ photon), competitiveness index (unitless), competition pressure (m^2^ plant^-1^), leaf area (m^2^ plant^-1^), plant height (m) and residual from the regression lines in Figure 7B (ε*_RUE_*, g fresh biomass mol^-1^ photon) for the Exp. winter and spring 2013 and spring 2016.

| Experiment | Traits | *H*^2^ | min | max | mean |
| --- | --- | --- | --- | --- | --- |
| Winter 2013 | Fresh biomass | 0.60 | 212 | 522 | 335±53 |
|  | RIE | 0.52 | 0.29 | 0.71 | 0.50±0.07 |
|  | RUE | 0.01 | 12.09 | 25.33 | 17.89±2.03 |
|  | Competitiveness index | 0.67 | 0.72 | 1.31 | 1.00±0.10 |
|  | Competition pressure | 0.00 | 0.28 | 0.41 | 0.34±0.02 |
|  | Leaf area | 0.52 | 0.20 | 0.45 | 0.32±0.04 |
|  | Plant height | 0.72 | 0.91 | 1.60 | 1.22±0.13 |
|  | ε*_RUE_* | 0.17 | -0.12 | 0.10 | 0.00±0.03 |
| Spring 2013 | Biomass | 0.52 | 263 | 625 | 413±64 |
|  | RIE | 0.54 | 0.40 | 1.24 | 0.78±0.14 |
|  | RUE | 0.13 | 9.47 | 19.29 | 15.02±1.56 |
|  | Competitiveness index | 0.67 | 0.53 | 1.40 | 0.99±0.15 |
|  | Competition pressure | 0.00 | 0.27 | 0.47 | 0.39±0.04 |
|  | Leaf area | 0.69 | 0.32 | 0.62 | 0.46±0.05 |
|  | Plant height | 0.64 | 1.05 | 2.13 | 1.48±0.14 |
|  | ε*_RUE_* | 0.25 | -0.13 | 0.08 | 0.00±0.03 |
| Spring 2016 | Biomass | 0.42 | 163 | 445 | 300±47 |
|  | RIE | 0.42 | 0.29 | 0.81 | 0.54±0.10 |
|  | RUE | 0.17 | 12.02 | 24.78 | 18.33±2.17 |
|  | Competitiveness index | 0.58 | 0.60 | 1.47 | 1.00±0.15 |
|  | Competition pressure | 0.04 | 0.25 | 0.46 | 0.36±0.04 |
|  | Leaf area | 0.41 | 0.27 | 0.53 | 0.40±0.04 |
|  | Plant height | 0.65 | 0.93 | 1.80 | 1.29±0.13 |
|  | ε*_RUE_* | 0.42 | -0.08 | 0.11 | 0.00±0.03 |

Table S2: QTLs of traits detected from the three experiments

| SNP Name^a^ | SNP position (pb) ^b^ | Chr^c^ | Bin^c^ | Experiment | Trait^d^ | cQTL^e^ | Region (cM)^f^ | Region (cM)^f^ | Region (Mbp)^f^ | Region (Mbp)^f^ | -log_10_(*P*) | R2 | Allelic effect ^g^ | MAF^h^ |
| --- | --- | --- | --- | --- | --- | --- | --- | --- | --- | --- | --- | --- | --- | --- |
| AX-90722479 | 275199428 | 1 | 1.1 | Spring_2013 | ε*_RUE_* | 1_1 | 224.27 | 224.56 | 275.00 | 275.31 | 5.56 | 0.04 | -0.01 | 0.12 |
| AX-90722493 | 275102950 | 1 | 1.1 | Spring_2013 | Biomass | 1_1 | 224.27 | 224.47 | 275.00 | 275.21 | 5.72 | 0.10 | -18.95 | 0.12 |
| S1_233449715 | 233449715 | 1 | 1.08 | Spring_2013 | LA | 1_2 | 192.61 | 192.81 | 233.37 | 233.54 | 5.29 | 0.04 | 0.02 | 0.11 |
| S1_233449730 | 233449730 | 1 | 1.08 | Spring_2013 | RIE_plant_CP_ | 1_2 | 192.61 | 192.81 | 233.37 | 233.54 | 5.53 | 0.05 | 0.04 | 0.12 |
| S1_19978587 | 19978587 | 1 | 1.02 | Spring_2013 | RIE | 1_3 | 47.05 | 47.25 | 19.94 | 20.03 | 5.63 | 0.06 | 0.04 | 0.09 |
| S1_19978587 | 19978587 | 1 | 1.02 | Spring_2013 | RIE_plant | 1_3 | 47.05 | 47.25 | 19.94 | 20.03 | 5.07 | 0.05 | 0.10 | 0.09 |
| S1_19978587 | 19978587 | 1 | 1.02 | Spring_2013 | RIE_plant_CP_ | 1_3 | 47.05 | 47.25 | 19.94 | 20.03 | 5.10 | 0.05 | 0.03 | 0.09 |
| AX-90724228 | 281337665 | 1 | 1.1 | Spring_2013 | RIE | 1_4 | 231.49 | 231.78 | 281.26 | 281.47 | 5.50 | 0.05 | 0.05 | 0.23 |
| AX-90724228 | 281337665 | 1 | 1.1 | Spring_2013 | RIE_plant | 1_4 | 231.51 | 231.78 | 281.27 | 281.47 | 6.30 | 0.07 | 0.12 | 0.23 |
| AX-91396339 | 281646863 | 1 | 1.1 | Spring_2013 | RIE | 1_5 | 231.95 | 232.15 | 281.59 | 281.72 | 5.52 | 0.05 | 0.04 | 0.19 |
| AX-91396339 | 281646863 | 1 | 1.1 | Spring_2013 | RIE_plant | 1_5 | 231.95 | 232.15 | 281.59 | 281.72 | 5.28 | 0.06 | 0.10 | 0.19 |
| AX-90577776 | 21759296 | 1 | 1.02 | Spring_2013 | RIE_plant | 1_6 | 50.43 | 50.63 | 21.71 | 21.82 | 5.70 | 0.06 | 0.11 | 0.42 |
| AX-90577776 | 21759296 | 1 | 1.02 | Spring_2013 | RIE_plant_CP_ | 1_6 | 50.43 | 50.63 | 21.71 | 21.82 | 5.04 | 0.05 | 0.04 | 0.42 |
| AX-90709464 | 227592531 | 1 | 1.07 | Spring_2016 | LA | 1_7 | 184.30 | 184.50 | 227.53 | 227.66 | 5.15 | 0.04 | 0.01 | 0.29 |
| PZE-101182889 | 227593940 | 1 | 1.07 | Spring_2016 | RIE_plant_CP_ | 1_7 | 184.30 | 184.50 | 227.53 | 227.66 | 5.22 | 0.05 | 0.03 | 0.30 |
| S1_217287980 | 217287980 | 1 | 1.07 | Winter_2013 | RIE_plant | 1_8 | 169.00 | 169.20 | 217.22 | 217.37 | 6.08 | 0.05 | 0.07 | 0.15 |
| S1_217287980 | 217287980 | 1 | 1.07 | Winter_2013 | RIE_plant_CP_ | 1_8 | 169.00 | 169.20 | 217.22 | 217.37 | 5.71 | 0.04 | 0.02 | 0.15 |
| SYN12337 | 7347483 | 1 | 1.01 | Winter_2013 | max_Height | NA | 16.40 | 16.60 | 7.31 | 7.40 | 5.03 | 0.10 | -40.95 | 0.25 |
| S1_8030757 | 8030757 | 1 | 1.01 | Winter_2013 | max_Height | NA | 18.01 | 18.21 | 7.99 | 8.08 | 5.01 | 0.10 | 36.47 | 0.07 |
| PZE-101035341 | 23054862 | 1 | 1.02 | Spring_2016 | RUE | NA | 52.63 | 52.83 | 22.99 | 23.12 | 5.31 | 0.07 | -0.64 | 0.43 |
| AX-91393729 | 25633900 | 1 | 1.02 | Spring_2016 | max_Height | NA | 56.46 | 56.66 | 25.56 | 25.71 | 5.11 | 0.10 | -38.05 | 0.20 |
| AX-90666390 | 59960241 | 1 | 1.04 | Winter_2013 | max_Height | NA | 75.34 | 75.54 | 59.70 | 60.23 | 7.74 | 0.13 | 48.23 | 0.42 |
| AX-91468155 | 79998859 | 1 | 1.04 | Spring_2013 | RIE_plant_CP_ | NA | 87.17 | 87.37 | 79.91 | 80.10 | 5.04 | 0.04 | -0.03 | 0.47 |
| SYN2036 | 112592216 | 1 | 1.05 | Winter_2013 | CI | NA | 113.11 | 113.31 | 111.97 | 113.25 | 5.12 | 0.05 | -0.03 | 0.49 |
| AX-90689028 | 149215068 | 1 | 1.05 | Spring_2013 | max_Height | NA | 115.53 | 115.73 | 148.82 | 149.60 | 5.32 | 0.10 | 41.82 | 0.19 |
| AX-90689578 | 151233480 | 1 | 1.05 | Spring_2013 | max_Height | NA | 116.14 | 116.34 | 150.94 | 151.52 | 5.13 | 0.10 | 42.23 | 0.22 |
| AX-91482189 | 155932252 | 1 | 1.05 | Spring_2013 | Biomass | NA | 118.20 | 118.40 | 155.74 | 156.12 | 5.03 | 0.09 | 18.34 | 0.05 |
| PZE-101129057 | 164556608 | 1 | 1.05 | Spring_2013 | LA | NA | 123.50 | 123.70 | 164.41 | 164.72 | 5.61 | 0.05 | 0.02 | 0.25 |
| S1_199502652 | 199502652 | 1 | 1.07 | Spring_2016 | RIE_plant | NA | 151.07 | 151.27 | 199.39 | 199.62 | 5.14 | 0.05 | 0.09 | 0.49 |
| PUT-163a-13389114-224 | 226177924 | 1 | 1.07 | Spring_2016 | RIE_plant_CP_ | NA | 182.08 | 182.29 | 226.11 | 226.25 | 5.18 | 0.05 | 0.03 | 0.07 |
| AX-90710265 | 230205831 | 1 | 1.08 | Spring_2016 | RIE_plant_CP_ | NA | 188.24 | 188.44 | 230.15 | 230.28 | 5.04 | 0.04 | 0.03 | 0.07 |
| S1_230622403 | 230622403 | 1 | 1.08 | Spring_2016 | RIE_plant_CP_ | NA | 188.82 | 189.02 | 230.55 | 230.70 | 5.94 | 0.06 | 0.03 | 0.06 |
| S1_230887240 | 230887240 | 1 | 1.08 | Spring_2013 | RIE_plant_CP_ | NA | 189.21 | 189.41 | 230.82 | 230.97 | 5.17 | 0.05 | 0.03 | 0.10 |
| AX-91496224 | 231073770 | 1 | 1.08 | Spring_2016 | RIE_plant_CP_ | NA | 189.46 | 189.69 | 231.00 | 231.17 | 5.58 | 0.05 | 0.03 | 0.12 |
| AX-91198148 | 276939370 | 1 | 1.1 | Spring_2013 | RIE_plant | NA | 226.10 | 226.30 | 276.85 | 277.04 | 5.17 | 0.06 | 0.10 | 0.08 |
| AX-91505034 | 279273362 | 1 | 1.1 | Winter_2013 | RIE_plant | NA | 228.76 | 228.96 | 279.19 | 279.36 | 5.02 | 0.04 | 0.06 | 0.17 |
| S1_292677821 | 292677821 | 1 | 1.11 | Winter_2013 | RUE | NA | 251.51 | 251.71 | 292.63 | 292.74 | 5.67 | 0.08 | -0.60 | 0.11 |
| PZE-101248001 | 293327617 | 1 | 1.11 | Winter_2013 | RUE | NA | 252.75 | 252.99 | 293.28 | 293.41 | 5.68 | 0.07 | 0.60 | 0.44 |
| AX-90739306 | 32941140 | 2 | 2.04 | Spring_2013 | LA | 2_1 | 74.42 | 74.63 | 32.80 | 33.10 | 5.48 | 0.05 | -0.02 | 0.14 |
| S2_33004586 | 33004586 | 2 | 2.04 | Spring_2016 | ε*_RUE_* | 2_1 | 74.46 | 74.66 | 32.86 | 33.15 | 5.40 | 0.04 | 0.01 | 0.45 |
| S2_9315284 | 9315284 | 2 | 2.02 | Spring_2016 | CI | NA | 32.34 | 32.54 | 9.29 | 9.36 | 5.11 | 0.05 | -0.04 | 0.18 |
| S2_16546989 | 16546989 | 2 | 2.03 | Winter_2013 | RIE_plant | NA | 53.85 | 54.05 | 16.52 | 16.59 | 5.11 | 0.05 | 0.06 | 0.12 |
| S2_21008126 | 21008126 | 2 | 2.03 | Winter_2013 | Biomass | NA | 63.29 | 63.49 | 20.96 | 21.07 | 5.19 | 0.09 | 15.36 | 0.07 |
| AX-91513306 | 21788191 | 2 | 2.03 | Spring_2013 | max_Height | NA | 64.55 | 64.75 | 21.73 | 21.86 | 6.13 | 0.11 | 46.51 | 0.24 |
| S2_28642805 | 28642805 | 2 | 2.04 | Spring_2016 | RUE | NA | 71.39 | 71.59 | 28.51 | 28.78 | 5.19 | 0.07 | 0.63 | 0.05 |
| S2_109105637 | 109105637 | 2 | 2.05 | Spring_2016 | CI | NA | 94.89 | 95.09 | 84.45 | 132.54 | 5.50 | 0.05 | -0.04 | 0.29 |
| AX-91411748 | 220894747 | 2 | 2.08 | Spring_2016 | ε*_RUE_* | NA | 179.25 | 179.50 | 220.84 | 220.98 | 5.27 | 0.04 | -0.01 | 0.37 |
| AX-90853995 | 227357679 | 3 | 3.09 | Spring_2013 | RIE | 3_1 | 178.34 | 178.54 | 227.32 | 227.41 | 5.37 | 0.05 | 0.04 | 0.16 |
| AX-90853995 | 227357679 | 3 | 3.09 | Spring_2013 | RIE_plant | 3_1 | 178.34 | 178.54 | 227.32 | 227.41 | 6.05 | 0.07 | 0.10 | 0.16 |
| AX-90853995 | 227357679 | 3 | 3.09 | Spring_2013 | RIE_plant_CP_ | 3_1 | 178.34 | 178.54 | 227.32 | 227.41 | 5.01 | 0.04 | 0.03 | 0.16 |
| S3_8270639 | 8270639 | 3 | 3.02 | Spring_2016 | CI | 3_2 | 31.08 | 31.28 | 8.25 | 8.30 | 5.12 | 0.05 | -0.04 | 0.09 |
| S3_8270639 | 8270639 | 3 | 3.02 | Spring_2016 | RIE_plant | 3_2 | 31.08 | 31.28 | 8.25 | 8.30 | 5.04 | 0.05 | -0.09 | 0.09 |
| S3_8270639 | 8270639 | 3 | 3.02 | Spring_2016 | RIE_plant_CP_ | 3_2 | 31.08 | 31.28 | 8.25 | 8.30 | 5.03 | 0.04 | -0.02 | 0.09 |
| AX-91595079 | 222483085 | 3 | 3.09 | Winter_2013 | LA | 3_3 | 168.37 | 168.57 | 222.44 | 222.53 | 5.63 | 0.05 | -0.01 | 0.20 |
| AX-91595079 | 222483085 | 3 | 3.09 | Winter_2013 | RIE_plant_CP_ | 3_3 | 168.37 | 168.57 | 222.44 | 222.53 | 5.46 | 0.05 | -0.02 | 0.20 |
| S3_7204839 | 7204839 | 3 | 3.02 | Spring_2016 | Biomass | NA | 27.36 | 27.56 | 7.18 | 7.23 | 5.88 | 0.10 | 15.22 | 0.17 |
| S3_19784941 | 19784941 | 3 | 3.04 | Spring_2013 | ε*_RUE_* | NA | 51.56 | 51.76 | 19.61 | 19.97 | 5.35 | 0.04 | -0.01 | 0.13 |
| S3_26309182 | 26309182 | 3 | 3.04 | Spring_2013 | max_Height | NA | 53.74 | 53.94 | 25.83 | 26.84 | 5.06 | 0.10 | 41.24 | 0.31 |
| AX-91584441 | 165469935 | 3 | 3.05 | Spring_2013 | RUE | NA | 80.33 | 80.53 | 165.41 | 165.54 | 5.27 | 0.07 | 0.44 | 0.31 |
| S3_187432605 | 187432605 | 3 | 3.06 | Spring_2013 | RUE | NA | 117.88 | 118.08 | 187.34 | 187.53 | 5.18 | 0.07 | 0.44 | 0.39 |
| S3_222671554 | 222671554 | 3 | 3.09 | Winter_2013 | ε*_RUE_* | NA | 168.75 | 168.95 | 222.63 | 222.72 | 5.26 | 0.04 | -0.01 | 0.13 |
| AX-91421745 | 234714245 | 4 | 4.09 | Spring_2013 | Biomass | 4_1 | 133.68 | 133.90 | 234.67 | 234.77 | 5.36 | 0.10 | 19.79 | 0.14 |
| S4_234717476 | 234717476 | 4 | 4.09 | Spring_2013 | LA | 4_1 | 133.70 | 133.90 | 234.68 | 234.77 | 5.52 | 0.05 | 0.02 | 0.09 |
| S4_234717476 | 234717476 | 4 | 4.09 | Spring_2013 | RIE | 4_1 | 133.70 | 133.90 | 234.68 | 234.77 | 6.11 | 0.06 | 0.05 | 0.09 |
| S4_234717476 | 234717476 | 4 | 4.09 | Spring_2013 | RIE_plant | 4_1 | 133.70 | 133.90 | 234.68 | 234.77 | 5.84 | 0.07 | 0.11 | 0.09 |
| S4_234717476 | 234717476 | 4 | 4.09 | Spring_2013 | RIE_plant_CP_ | 4_1 | 133.70 | 133.90 | 234.68 | 234.77 | 5.13 | 0.05 | 0.03 | 0.09 |
| S4_234722319 | 234722319 | 4 | 4.09 | Spring_2016 | Biomass | 4_1 | 133.70 | 133.90 | 234.68 | 234.77 | 5.83 | 0.10 | -14.44 | 0.23 |
| S4_234722319 | 234722319 | 4 | 4.09 | Spring_2016 | CI | 4_1 | 133.70 | 133.90 | 234.68 | 234.77 | 5.46 | 0.05 | -0.04 | 0.23 |
| S4_234722319 | 234722319 | 4 | 4.09 | Spring_2016 | RIE | 4_1 | 133.70 | 133.90 | 234.68 | 234.77 | 6.83 | 0.07 | -0.03 | 0.23 |
| S4_234722319 | 234722319 | 4 | 4.09 | Spring_2016 | RIE_plant | 4_1 | 133.70 | 133.90 | 234.68 | 234.77 | 7.88 | 0.10 | -0.11 | 0.23 |
| S4_234722319 | 234722319 | 4 | 4.09 | Spring_2016 | RIE_plant_CP_ | 4_1 | 133.70 | 133.90 | 234.68 | 234.77 | 6.09 | 0.06 | -0.03 | 0.23 |
| S4_133339715 | 133339715 | 4 | 4.05 | Spring_2013 | LA | 4_2 | 54.89 | 55.09 | 131.71 | 134.73 | 5.67 | 0.05 | 0.02 | 0.15 |
| S4_133339715 | 133339715 | 4 | 4.05 | Spring_2013 | RIE_plant_CP_ | 4_2 | 54.89 | 55.09 | 131.71 | 134.73 | 5.38 | 0.05 | 0.03 | 0.15 |
| AX-90892474 | 140557398 | 4 | 4.05 | Spring_2013 | LA | 4_3 | 55.68 | 55.94 | 139.79 | 141.16 | 5.58 | 0.05 | 0.02 | 0.10 |
| AX-90892474 | 140557398 | 4 | 4.05 | Spring_2013 | RIE | 4_3 | 55.72 | 55.92 | 140.02 | 141.07 | 5.12 | 0.05 | 0.04 | 0.10 |
| AX-90892474 | 140557398 | 4 | 4.05 | Spring_2013 | RIE_plant_CP_ | 4_3 | 55.72 | 55.92 | 140.02 | 141.07 | 5.08 | 0.04 | 0.03 | 0.10 |
| AX-90898104 | 161548767 | 4 | 4.06 | Winter_2013 | Biomass | 4_4 | 70.29 | 70.49 | 161.48 | 161.63 | 5.92 | 0.09 | 16.07 | 0.08 |
| AX-90898104 | 161548767 | 4 | 4.06 | Winter_2013 | RIE | 4_4 | 70.29 | 70.49 | 161.48 | 161.63 | 5.08 | 0.04 | 0.02 | 0.08 |
| S4_236976065 | 236976065 | 4 | 4.1 | Winter_2013 | Biomass | 4_5 | 139.33 | 139.53 | 236.95 | 237.02 | 5.42 | 0.10 | -16.45 | 0.07 |
| S4_236976065 | 236976065 | 4 | 4.1 | Winter_2013 | LA | 4_5 | 139.33 | 139.53 | 236.95 | 237.02 | 5.66 | 0.05 | -0.01 | 0.07 |
| AX-91414814 | 238878021 | 4 | 4.1 | Winter_2013 | RIE_plant | 4_6 | 144.08 | 144.28 | 238.84 | 238.93 | 5.42 | 0.05 | 0.06 | 0.34 |
| AX-91414814 | 238878021 | 4 | 4.1 | Winter_2013 | RIE_plant_CP_ | 4_6 | 144.08 | 144.28 | 238.84 | 238.93 | 5.31 | 0.04 | 0.02 | 0.34 |
| AX-90859348 | 14029351 | 4 | 4.03 | Spring_2016 | RIE_plant | NA | 34.51 | 34.71 | 13.98 | 14.09 | 5.33 | 0.06 | -0.09 | 0.27 |
| S4_17321927 | 17321927 | 4 | 4.03 | Spring_2016 | RIE | NA | 39.54 | 39.74 | 17.25 | 17.40 | 5.01 | 0.04 | -0.03 | 0.27 |
| S4_18695379 | 18695379 | 4 | 4.03 | Winter_2013 | RIE | NA | 41.21 | 41.41 | 18.62 | 18.80 | 5.15 | 0.04 | -0.02 | 0.08 |
| S4_18911894 | 18911894 | 4 | 4.03 | Winter_2013 | RIE | NA | 41.44 | 41.64 | 18.82 | 19.01 | 5.17 | 0.04 | -0.02 | 0.11 |
| AX-90607009 | 35211747 | 4 | 4.05 | Spring_2013 | LA | NA | 49.64 | 49.84 | 34.88 | 35.55 | 5.22 | 0.04 | 0.02 | 0.15 |
| AX-91219283 | 96199251 | 4 | 4.05 | Winter_2013 | RIE | NA | 54.43 | 54.66 | 91.29 | 126.57 | 6.23 | 0.05 | 0.02 | 0.34 |
| S4_144532017 | 144532017 | 4 | 4.05 | Spring_2016 | RUE | NA | 56.72 | 56.92 | 144.22 | 144.84 | 5.43 | 0.07 | 0.63 | 0.06 |
| AX-91627416 | 162926547 | 4 | 4.06 | Winter_2013 | LA | NA | 72.19 | 72.39 | 162.86 | 163.01 | 5.51 | 0.03 | 0.01 | 0.12 |
| AX-91383497 | 179661722 | 4 | 4.07 | Winter_2013 | RUE | NA | 99.03 | 99.23 | 179.60 | 179.73 | 5.12 | 0.07 | 0.57 | 0.17 |
| AX-90904094 | 184154501 | 4 | 4.08 | Winter_2013 | max_Height | NA | 105.70 | 105.90 | 184.08 | 184.23 | 5.04 | 0.09 | -38.75 | 0.49 |
| AX-90618274 | 226938367 | 4 | 4.09 | Spring_2016 | RUE | NA | 119.15 | 119.35 | 226.86 | 227.03 | 5.01 | 0.06 | 0.60 | 0.06 |
| SYNGENTA16214 | 229180335 | 4 | 4.09 | Spring_2013 | Biomass | NA | 122.35 | 122.55 | 229.12 | 229.25 | 5.16 | 0.09 | -19.04 | 0.24 |
| AX-91448384 | 185413686 | 5 | 5.05 | Spring_2013 | CI | 5_1 | 116.25 | 116.45 | 185.32 | 185.51 | 5.38 | 0.05 | -0.04 | 0.31 |
| AX-91448384 | 185413686 | 5 | 5.05 | Spring_2013 | RIE_plant | 5_1 | 116.25 | 116.55 | 185.32 | 185.60 | 6.06 | 0.07 | -0.10 | 0.31 |
| AX-91681222 | 213943752 | 5 | 5.08 | Spring_2013 | RIE | 5_2 | 186.31 | 186.65 | 213.90 | 213.98 | 6.38 | 0.07 | -0.05 | 0.12 |
| S5_213944990 | 213944990 | 5 | 5.08 | Spring_2013 | LA | 5_2 | 186.40 | 186.60 | 213.92 | 213.97 | 5.03 | 0.04 | -0.02 | 0.13 |
| S5_213945086 | 213945086 | 5 | 5.08 | Spring_2013 | RIE_plant | 5_2 | 186.45 | 186.65 | 213.93 | 213.98 | 5.14 | 0.06 | -0.10 | 0.16 |
| AX-91422789 | 12229763 | 5 | 5.02 | Spring_2013 | LA | 5_3 | 37.17 | 37.39 | 12.18 | 12.28 | 5.14 | 0.04 | 0.02 | 0.28 |
| AX-91436202 | 12215772 | 5 | 5.02 | Spring_2013 | RIE_plant_CP_ | 5_3 | 37.17 | 37.37 | 12.18 | 12.27 | 5.24 | 0.05 | -0.03 | 0.20 |
| AX-91439009 | 4469305 | 5 | 5.01 | Spring_2013 | RIE_plant | 5_4 | 16.23 | 16.43 | 4.45 | 4.50 | 5.37 | 0.06 | 0.10 | 0.11 |
| S5_4474778 | 4474778 | 5 | 5.01 | Spring_2016 | Biomass | 5_4 | 16.23 | 16.43 | 4.45 | 4.50 | 5.08 | 0.08 | 13.71 | 0.36 |
| AX-90976610 | 212681353 | 5 | 5.08 | Spring_2016 | Biomass | 5_5 | 180.58 | 180.78 | 212.66 | 212.71 | 6.29 | 0.11 | -14.48 | 0.09 |
| AX-90976610 | 212681353 | 5 | 5.08 | Spring_2016 | RIE | 5_5 | 180.58 | 180.78 | 212.66 | 212.71 | 6.10 | 0.06 | -0.03 | 0.09 |
| AX-90976610 | 212681353 | 5 | 5.08 | Spring_2016 | RIE_plant | 5_5 | 180.58 | 180.78 | 212.66 | 212.71 | 6.23 | 0.07 | -0.09 | 0.09 |
| AX-90976610 | 212681353 | 5 | 5.08 | Spring_2016 | RIE_plant_CP_ | 5_5 | 180.58 | 180.78 | 212.66 | 212.71 | 5.71 | 0.05 | -0.03 | 0.09 |
| AX-91673524 | 172289369 | 5 | 5.04 | Spring_2016 | CI | 5_6 | 99.70 | 99.90 | 172.23 | 172.36 | 5.22 | 0.05 | 0.04 | 0.46 |
| AX-91673524 | 172289369 | 5 | 5.04 | Spring_2016 | RIE_plant_CP_ | 5_6 | 99.70 | 99.90 | 172.23 | 172.36 | 5.01 | 0.04 | 0.03 | 0.46 |
| S5_3393737 | 3393737 | 5 | 5.01 | Winter_2013 | Biomass | NA | 12.39 | 12.59 | 3.37 | 3.42 | 5.46 | 0.09 | 15.77 | 0.09 |
| AX-90922252 | 6278530 | 5 | 5.01 | Spring_2013 | RIE_plant | NA | 22.23 | 22.43 | 6.25 | 6.32 | 5.18 | 0.06 | 0.11 | 0.07 |
| AX-90924100 | 13138837 | 5 | 5.02 | Spring_2016 | LA | NA | 39.15 | 39.35 | 13.10 | 13.19 | 5.85 | 0.05 | 0.01 | 0.46 |
| AX-91644700 | 15544264 | 5 | 5.03 | Spring_2016 | LA | NA | 43.83 | 44.03 | 15.49 | 15.60 | 5.16 | 0.04 | 0.01 | 0.28 |
| AX-90634057 | 16432030 | 5 | 5.03 | Spring_2016 | LA | NA | 45.35 | 45.55 | 16.37 | 16.50 | 5.16 | 0.04 | 0.01 | 0.40 |
| AX-90929252 | 31418786 | 5 | 5.03 | Spring_2016 | LA | NA | 56.58 | 56.78 | 30.92 | 31.96 | 5.55 | 0.04 | 0.01 | 0.20 |
| S5_35113319 | 35113319 | 5 | 5.03 | Spring_2016 | LA | NA | 57.16 | 57.36 | 34.40 | 35.86 | 5.08 | 0.04 | 0.01 | 0.45 |
| S5_150934216 | 150934216 | 5 | 5.04 | Spring_2016 | RIE_plant_CP_ | NA | 82.08 | 82.28 | 150.42 | 151.43 | 5.74 | 0.05 | -0.03 | 0.26 |
| AX-91672005 | 164220786 | 5 | 5.04 | Spring_2016 | RIE | NA | 88.96 | 89.16 | 164.13 | 164.32 | 5.18 | 0.03 | 0.03 | 0.34 |
| AX-91409179 | 174291278 | 5 | 5.05 | Spring_2016 | RIE_plant_CP_ | NA | 102.66 | 102.88 | 174.23 | 174.37 | 5.25 | 0.05 | -0.03 | 0.11 |
| SYN9631 | 179928870 | 5 | 5.05 | Spring_2016 | CI | NA | 110.33 | 110.53 | 179.85 | 180.02 | 5.40 | 0.05 | -0.04 | 0.45 |
| AX-91675720 | 184349189 | 5 | 5.05 | Spring_2013 | CI | NA | 114.92 | 115.31 | 184.08 | 184.45 | 5.79 | 0.06 | -0.05 | 0.34 |
| AX-90970256 | 189909183 | 5 | 5.05 | Winter_2013 | LA | NA | 121.62 | 121.99 | 189.84 | 190.12 | 5.97 | 0.05 | 0.01 | 0.15 |
| AX-91678621 | 200318714 | 5 | 5.06 | Winter_2013 | RUE | NA | 137.54 | 137.74 | 200.28 | 200.37 | 5.27 | 0.07 | -0.57 | 0.44 |
| S5_202095840 | 202095840 | 5 | 5.06 | Spring_2016 | LA | NA | 141.76 | 141.96 | 202.07 | 202.14 | 5.41 | 0.04 | 0.01 | 0.39 |
| S5_202298957 | 202298957 | 5 | 5.06 | Spring_2016 | LA | NA | 142.28 | 142.48 | 202.27 | 202.34 | 5.39 | 0.04 | -0.01 | 0.09 |
| AX-90974195 | 204172827 | 5 | 5.06 | Spring_2016 | LA | NA | 147.57 | 147.77 | 204.14 | 204.21 | 6.19 | 0.06 | -0.01 | 0.09 |
| S5_214053489 | 214053489 | 5 | 5.08 | Spring_2013 | RIE | NA | 186.91 | 187.11 | 214.03 | 214.08 | 6.27 | 0.07 | -0.04 | 0.09 |
| S6_33623678 | 33623678 | 6 | 6.01 | Spring_2016 | Biomass | 6_1 | 12.18 | 12.38 | 33.06 | 34.21 | 6.32 | 0.09 | 14.66 | 0.18 |
| S6_33623678 | 33623678 | 6 | 6.01 | Spring_2016 | RIE | 6_1 | 12.18 | 12.38 | 33.06 | 34.21 | 5.17 | 0.04 | 0.03 | 0.18 |
| S6_33623678 | 33623678 | 6 | 6.01 | Spring_2016 | RIE_plant | 6_1 | 12.18 | 12.38 | 33.06 | 34.21 | 5.09 | 0.05 | 0.09 | 0.18 |
| AX-90985936 | 34217148 | 6 | 6.01 | Spring_2016 | RIE_plant_CP_ | 6_1 | 12.18 | 12.49 | 33.06 | 34.86 | 5.03 | 0.04 | 0.03 | 0.45 |
| AX-90636354 | 31869518 | 6 | 6.01 | Spring_2016 | LA | 6_2 | 11.86 | 12.06 | 31.40 | 32.39 | 5.12 | 0.04 | -0.01 | 0.50 |
| AX-91687108 | 32274766 | 6 | 6.01 | Spring_2016 | Biomass | 6_2 | 11.94 | 12.16 | 31.77 | 32.94 | 5.11 | 0.08 | 13.32 | 0.23 |
| S6_2006822 | 2006822 | 6 | 6 | Spring_2016 | RIE_plant | NA | 1.60 | 1.80 | 1.91 | 2.12 | 5.18 | 0.04 | 0.09 | 0.38 |
| AX-91686078 | 26816708 | 6 | 6.01 | Spring_2016 | LA | NA | 10.13 | 10.40 | 26.47 | 27.16 | 5.08 | 0.04 | -0.01 | 0.26 |
| PZE-106017013 | 40053175 | 6 | 6.01 | Spring_2016 | LA | NA | 12.69 | 12.89 | 36.60 | 53.63 | 5.11 | 0.04 | -0.01 | 0.15 |
| AX-90994344 | 67294188 | 6 | 6.01 | Spring_2016 | LA | NA | 13.68 | 14.03 | 60.80 | 68.24 | 6.46 | 0.06 | -0.01 | 0.15 |
| SYN4001 | 157820229 | 6 | 6.06 | Spring_2016 | RIE_plant_CP_ | NA | 87.86 | 88.06 | 157.79 | 157.86 | 5.09 | 0.04 | -0.02 | 0.34 |
| AX-91047921 | 100351915 | 7 | 7.02 | Spring_2013 | LA | 7_1 | 41.20 | 41.59 | 99.84 | 101.31 | 5.80 | 0.05 | -0.02 | 0.13 |
| AX-91047921 | 100351915 | 7 | 7.02 | Spring_2013 | max_Height | 7_1 | 41.20 | 41.70 | 99.84 | 101.65 | 6.27 | 0.12 | -46.47 | 0.13 |
| AX-91047921 | 100351915 | 7 | 7.02 | Spring_2013 | RIE_plant_CP_ | 7_1 | 41.20 | 41.70 | 99.84 | 101.65 | 6.87 | 0.07 | -0.04 | 0.13 |
| AX-91047921 | 100351915 | 7 | 7.02 | Spring_2013 | CI | 7_1 | 41.22 | 41.70 | 99.93 | 101.65 | 6.63 | 0.07 | -0.05 | 0.13 |
| AX-91047921 | 100351915 | 7 | 7.02 | Spring_2013 | RIE_plant_CP_ | 7_1 | 41.22 | 41.42 | 99.96 | 100.73 | 5.03 | 0.05 | -0.09 | 0.13 |
| S7_100887626 | 100887626 | 7 | 7.02 | Winter_2013 | Biomass | 7_1 | 41.37 | 41.57 | 100.53 | 101.24 | 5.28 | 0.09 | 15.27 | 0.36 |
| AX-91048276 | 101578020 | 7 | 7.02 | Spring_2013 | RIE_plant | 7_1 | 41.58 | 41.78 | 101.26 | 101.89 | 5.48 | 0.06 | -0.09 | 0.49 |
| S7_96045091 | 96045091 | 7 | 7.02 | Spring_2013 | LA | 7_2 | 40.43 | 40.63 | 95.25 | 96.79 | 5.37 | 0.05 | -0.02 | 0.11 |
| S7_96045091 | 96045091 | 7 | 7.02 | Spring_2013 | RIE_plant_CP_ | 7_2 | 40.43 | 40.63 | 95.25 | 96.79 | 5.28 | 0.05 | -0.03 | 0.11 |
| AX-91047921 | 100351915 | 7 | 7.02 | Spring_2013 | LA | 7_3 | 41.20 | 41.59 | 99.84 | 101.31 | 5.80 | 0.05 | -0.02 | 0.13 |
| AX-91047921 | 100351915 | 7 | 7.02 | Spring_2013 | max_Height | 7_3 | 41.20 | 41.70 | 99.84 | 101.65 | 6.27 | 0.12 | -46.47 | 0.13 |
| AX-91047921 | 100351915 | 7 | 7.02 | Spring_2013 | RIE_plant_CP_ | 7_3 | 41.20 | 41.70 | 99.84 | 101.65 | 6.87 | 0.07 | -0.04 | 0.13 |
| AX-91047921 | 100351915 | 7 | 7.02 | Spring_2013 | CI | 7_3 | 41.22 | 41.70 | 99.93 | 101.65 | 6.63 | 0.07 | -0.05 | 0.13 |
| AX-91048276 | 101578020 | 7 | 7.02 | Spring_2013 | RIE_plant | 7_3 | 41.58 | 41.78 | 101.26 | 101.89 | 5.48 | 0.06 | -0.09 | 0.49 |
| AX-91047921 | 100351915 | 7 | 7.02 | Spring_2013 | LA | 7_4 | 41.20 | 41.59 | 99.84 | 101.31 | 5.80 | 0.05 | -0.02 | 0.13 |
| AX-91047921 | 100351915 | 7 | 7.02 | Spring_2013 | max_Height | 7_4 | 41.20 | 41.70 | 99.84 | 101.65 | 6.27 | 0.12 | -46.47 | 0.13 |
| AX-91047921 | 100351915 | 7 | 7.02 | Spring_2013 | RIE_plant_CP_ | 7_4 | 41.20 | 41.70 | 99.84 | 101.65 | 6.87 | 0.07 | -0.04 | 0.13 |
| AX-91047921 | 100351915 | 7 | 7.02 | Spring_2013 | CI | 7_4 | 41.22 | 41.70 | 99.93 | 101.65 | 6.63 | 0.07 | -0.05 | 0.13 |
| AX-91047921 | 100351915 | 7 | 7.02 | Spring_2013 | RIE_plant | 7_4 | 41.22 | 41.42 | 99.96 | 100.73 | 5.03 | 0.05 | -0.09 | 0.13 |
| S7_100887626 | 100887626 | 7 | 7.02 | Winter_2013 | Biomass | 7_4 | 41.37 | 41.57 | 100.53 | 101.24 | 5.28 | 0.09 | 15.27 | 0.36 |
| AX-90614946 | 164831971 | 7 | 7.04 | Spring_2016 | Biomass | 7_5 | 102.35 | 102.55 | 164.80 | 164.87 | 5.85 | 0.09 | 14.03 | 0.17 |
| S7_164832586 | 164832586 | 7 | 7.04 | Spring_2016 | RIE_plant | 7_5 | 102.35 | 102.55 | 164.80 | 164.87 | 5.18 | 0.05 | -0.09 | 0.50 |
| AX-91060270 | 147060237 | 7 | 7.03 | Winter_2013 | RIE | 7_6 | 85.20 | 85.40 | 146.48 | 147.78 | 5.48 | 0.05 | 0.02 | 0.18 |
| AX-91060270 | 147060237 | 7 | 7.03 | Winter_2013 | RIE_plant | 7_6 | 85.20 | 85.40 | 146.48 | 147.78 | 5.57 | 0.05 | 0.06 | 0.17 |
| AX-91739713 | 147056968 | 7 | 7.03 | Winter_2013 | RIE_plant_CP_ | 7_6 | 85.20 | 85.40 | 146.48 | 147.78 | 5.33 | 0.04 | 0.02 | 0.17 |
| AX-91739982 | 148465717 | 7 | 7.03 | Spring_2016 | RUE | 7_6 | 85.38 | 85.58 | 147.60 | 149.59 | 5.42 | 0.07 | -0.65 | 0.46 |
| AX-91741386 | 156477518 | 7 | 7.04 | Winter_2013 | Biomass | 7_7 | 87.34 | 87.54 | 156.34 | 156.62 | 5.62 | 0.08 | 15.48 | 0.32 |
| AX-91741386 | 156477518 | 7 | 7.04 | Winter_2013 | RIE_plant | 7_7 | 87.34 | 87.54 | 156.34 | 156.62 | 6.02 | 0.05 | 0.07 | 0.32 |
| AX-91413764 | 8177986 | 7 | 7.01 | Winter_2013 | RIE_plant | NA | 25.36 | 25.56 | 8.14 | 8.23 | 5.27 | 0.05 | 0.06 | 0.36 |
| S7_9198475 | 9198475 | 7 | 7.01 | Spring_2016 | Biomass | NA | 27.40 | 27.60 | 9.15 | 9.26 | 5.04 | 0.07 | 12.96 | 0.41 |
| PZE-107013677 | 10163248 | 7 | 7.01 | Spring_2016 | Biomass | NA | 29.02 | 29.22 | 10.10 | 10.23 | 5.55 | 0.10 | 14.10 | 0.11 |
| S7_21146088 | 21146088 | 7 | 7.02 | Spring_2016 | max_Height | NA | 36.08 | 36.28 | 20.78 | 21.54 | 5.28 | 0.10 | 40.71 | 0.27 |
| AX-91048647 | 103018426 | 7 | 7.02 | Winter_2013 | RIE_plant | NA | 42.08 | 42.28 | 102.76 | 103.28 | 5.04 | 0.05 | 0.06 | 0.14 |
| S7_107327325 | 107327325 | 7 | 7.02 | Spring_2016 | max_Height | NA | 43.91 | 44.11 | 107.09 | 107.59 | 5.12 | 0.10 | -38.94 | 0.48 |
| S7_113628572 | 113628572 | 7 | 7.02 | Spring_2013 | max_Height | NA | 45.73 | 45.93 | 113.31 | 113.95 | 6.07 | 0.11 | -47.55 | 0.13 |
| PZE-107063120 | 120257394 | 7 | 7.02 | Winter_2013 | ε*_RUE_* | NA | 49.35 | 49.55 | 120.16 | 120.37 | 5.33 | 0.04 | 0.01 | 0.36 |
| S7_142903704 | 142903704 | 7 | 7.03 | Spring_2016 | LA | NA | 83.92 | 84.12 | 142.71 | 143.11 | 5.22 | 0.04 | -0.01 | 0.32 |
| S7_157459663 | 157459663 | 7 | 7.04 | Spring_2016 | ε*_RUE_* | NA | 88.14 | 88.34 | 157.36 | 157.57 | 5.35 | 0.04 | 0.01 | 0.45 |
| AX-91742249 | 161129901 | 7 | 7.04 | Winter_2013 | RIE | NA | 93.29 | 93.49 | 161.08 | 161.19 | 6.94 | 0.07 | 0.02 | 0.19 |
| AX-91064612 | 162669036 | 7 | 7.04 | Spring_2016 | ε*_RUE_* | NA | 96.58 | 96.78 | 162.63 | 162.72 | 5.49 | 0.03 | -0.01 | 0.45 |
| S7_162977534 | 162977534 | 7 | 7.04 | Spring_2016 | max_Height | NA | 97.33 | 97.53 | 162.94 | 163.03 | 5.02 | 0.09 | 37.66 | 0.32 |
| S7_174441844 | 174441844 | 7 | 7.06 | Winter_2013 | LA | NA | 138.46 | 138.66 | 174.42 | 174.47 | 5.37 | 0.04 | 0.01 | 0.08 |
| AX-91776564 | 167709575 | 8 | 8.07 | Spring_2013 | LA | 8_1 | 155.52 | 155.72 | 167.68 | 167.75 | 5.32 | 0.04 | 0.02 | 0.06 |
| AX-91776564 | 167709575 | 8 | 8.07 | Spring_2013 | RIE | 8_1 | 155.52 | 155.72 | 167.68 | 167.75 | 5.68 | 0.06 | 0.04 | 0.06 |
| AX-91776564 | 167709575 | 8 | 8.07 | Spring_2013 | RIE_plant | 8_1 | 155.52 | 155.72 | 167.68 | 167.75 | 6.00 | 0.07 | 0.11 | 0.06 |
| AX-91776564 | 167709575 | 8 | 8.07 | Spring_2013 | RIE_plant_CP_ | 8_1 | 155.52 | 155.72 | 167.68 | 167.75 | 5.60 | 0.05 | 0.03 | 0.06 |
| S8_161526188 | 161526188 | 8 | 8.06 | Spring_2016 | RIE | 8_2 | 139.88 | 140.08 | 161.48 | 161.59 | 5.77 | 0.05 | 0.03 | 0.28 |
| S8_161526188 | 161526188 | 8 | 8.06 | Spring_2016 | RIE_plant_CP_ | 8_2 | 139.88 | 140.09 | 161.48 | 161.60 | 5.17 | 0.04 | 0.03 | 0.28 |
| S8_161544570 | 161544570 | 8 | 8.06 | Winter_2013 | RIE | 8_2 | 139.88 | 140.09 | 161.48 | 161.60 | 6.15 | 0.04 | 0.02 | 0.41 |
| S8_161544570 | 161544570 | 8 | 8.06 | Spring_2016 | Biomass | 8_2 | 139.89 | 140.09 | 161.49 | 161.60 | 5.54 | 0.09 | 14.21 | 0.41 |
| S8_161544570 | 161544570 | 8 | 8.06 | Winter_2013 | RIE_plant | 8_2 | 139.89 | 140.09 | 161.49 | 161.60 | 5.66 | 0.04 | 0.06 | 0.41 |
| S8_18350687 | 18350687 | 8 | 8.02 | Spring_2013 | max_Height | NA | 46.90 | 47.10 | 18.31 | 18.40 | 5.01 | 0.10 | 39.83 | 0.14 |
| AX-91752011 | 36570640 | 8 | 8.03 | Spring_2016 | RUE | NA | 64.24 | 64.44 | 35.91 | 37.22 | 5.01 | 0.07 | -0.60 | 0.40 |
| AX-91101801 | 127774161 | 8 | 8.05 | Spring_2016 | CI | NA | 113.84 | 114.04 | 127.72 | 127.83 | 5.42 | 0.05 | -0.05 | 0.39 |
| S8_159395811 | 159395811 | 8 | 8.06 | Spring_2013 | CI | NA | 136.55 | 136.75 | 159.33 | 159.48 | 5.46 | 0.05 | -0.05 | 0.16 |
| AX-91111574 | 162220695 | 8 | 8.06 | Spring_2016 | RIE_plant_CP_ | NA | 141.16 | 141.36 | 162.17 | 162.28 | 5.06 | 0.04 | -0.03 | 0.48 |
| S9_26630602 | 26630602 | 9 | 9.03 | Winter_2013 | ε*_RUE_* | NA | 56.86 | 57.06 | 26.38 | 26.91 | 5.46 | 0.04 | 0.01 | 0.19 |
| S9_73520507 | 73520507 | 9 | 9.03 | Spring_2013 | RIE | NA | 58.29 | 58.49 | 68.90 | 75.21 | 5.21 | 0.05 | 0.04 | 0.05 |
| S9_79717120 | 79717120 | 9 | 9.03 | Spring_2013 | RIE | NA | 58.70 | 58.90 | 78.14 | 81.83 | 5.05 | 0.05 | 0.04 | 0.10 |
| S9_90366933 | 90366933 | 9 | 9.03 | Winter_2013 | max_Height | NA | 59.05 | 59.27 | 88.04 | 91.83 | 5.55 | 0.10 | 40.85 | 0.18 |
| PZE-109061692 | 103236962 | 9 | 9.04 | Winter_2013 | max_Height | NA | 62.49 | 62.69 | 103.05 | 103.44 | 5.13 | 0.09 | -40.21 | 0.44 |
| S9_149546530 | 149546530 | 9 | 9.07 | Spring_2013 | LA | NA | 112.58 | 112.78 | 149.53 | 149.58 | 5.09 | 0.04 | -0.02 | 0.19 |
| AX-91158047 | 3275712 | 10 | 10.01 | Winter_2013 | RIE_plant | NA | 14.86 | 15.06 | 3.26 | 3.31 | 5.01 | 0.04 | 0.06 | 0.42 |
| AX-91158823 | 6370824 | 10 | 10.02 | Spring_2013 | ε*_RUE_* | NA | 27.31 | 27.51 | 6.35 | 6.40 | 5.77 | 0.05 | 0.01 | 0.10 |
| S10_10667775 | 10667775 | 10 | 10.02 | Spring_2013 | Biomass | NA | 38.03 | 38.23 | 10.62 | 10.73 | 5.45 | 0.09 | -18.27 | 0.41 |
| AX-91820335 | 70304876 | 10 | 10.03 | Spring_2016 | LA | NA | 46.28 | 46.48 | 18.59 | 71.23 | 5.00 | 0.03 | 0.01 | 0.13 |
| AX-91178436 | 81824590 | 10 | 10.03 | Winter_2013 | RUE | NA | 56.04 | 56.24 | 81.74 | 81.91 | 5.40 | 0.07 | 0.58 | 0.40 |
| S10_83456205 | 83456205 | 10 | 10.03 | Winter_2013 | RUE | NA | 58.25 | 58.45 | 83.40 | 83.53 | 5.45 | 0.07 | 0.59 | 0.41 |
| AX-91185915 | 109579822 | 10 | 10.04 | Winter_2013 | RIE_plant | NA | 75.77 | 75.97 | 109.10 | 110.03 | 5.03 | 0.05 | 0.06 | 0.08 |
| S10_114855805 | 114855805 | 10 | 10.04 | Spring_2013 | CI | NA | 77.44 | 77.67 | 114.45 | 115.21 | 5.06 | 0.05 | 0.05 | 0.11 |
| S10_117785247 | 117785247 | 10 | 10.04 | Winter_2013 | RUE | NA | 78.24 | 78.44 | 117.40 | 118.19 | 6.20 | 0.08 | 0.63 | 0.05 |
| AX-91833124 | 140862040 | 10 | 10.06 | Spring_2016 | LA | NA | 106.38 | 106.58 | 140.84 | 140.89 | 5.13 | 0.04 | -0.01 | 0.19 |
| PZE-110092504 | 140934708 | 10 | 10.06 | Spring_2016 | LA | NA | 106.67 | 106.87 | 140.91 | 140.96 | 5.25 | 0.04 | -0.01 | 0.19 |

^a^SNP with the highest -log_10_(*P*-value). ^b^SNP physical position. ^c^Chromosome and bin (a subdivision of the maize chromosomes). ^d^The abbreviation of the listed traits can be found in the manuscript and Table 1. ^e^Consensus QTL where QTLs of more traits were detected. ^f^ QTL region (in genetic and physical units). ^g^Effect of the allele of the reference hybrid B73. ^h^Minor allele frequency.
